# CRISPR-based functional genomics for dissecting therapeutic dependency in primary acute myeloid leukemia samples

**DOI:** 10.1016/j.molcel.2026.02.003

**Published:** 2026-02-26

**Authors:** Zhendong Cao, Sixiang Yu, Jacqueline Peng, Declan R. Barrett, Yuqiao Liu, Jonathan H. Sussman, Changya Chen, Anusha Thadi, Li Liu, Fatemeh Alikarami, Jason Xu, Martin P. Carroll, Kai Tan, Kathrin M. Bernt, Junwei Shi

**Affiliations:** 1 Department of Cancer Biology, Perelman School of Medicine, University of Pennsylvania, Philadelphia, PA 19104, USA; 2 Epigenetics Institute, Perelman School of Medicine, University of Pennsylvania, Philadelphia, PA 19104, USA; 3 Abramson Family Cancer Research Institute, Perelman School of Medicine, University of Pennsylvania, Philadelphia, PA 19104, USA; 4 Graduate Group in Cell and Molecular Biology, University of Pennsylvania, Philadelphia, PA, USA; 5 Graduate Group in Genomics and Computational Biology, University of Pennsylvania, Philadelphia, PA, USA; 6 Division of Oncology, Department of Pediatrics, University of Pennsylvania, Philadelphia, PA 19104, USA; 7 Center for Childhood Cancer Research, The Children’s Hospital of Philadelphia, Philadelphia, PA, USA; 8 Division of Hematology and Oncology, Perelman School of Medicine, University of Pennsylvania, Philadelphia, PA 19104, USA; 9 These authors contributed equally; 10 These authors contributed equally; 11 Lead contact

## Abstract

Cancer functional genomics enables high-throughput target discovery and mechanistic investigation, yet its application has remained largely confined to mouse models and established human cancer cell lines. Direct functional interrogation of heterogeneous primary tumors offers a powerful opportunity to evaluate therapeutic targets and uncover cancer dependencies or resistance mechanisms. Here, we developed an optimized CRISPR-based platform for functional genomics in patient-derived xenograft and primary acute myeloid leukemia (AML) samples harboring diverse pathogenic mutations. Integrated *in vitro* and *in vivo* CRISPR-Cas9 knockout and CRISPR interference (CRISPRi) dropout screens validated known AML-biased targets and identified *cis-*regulatory elements essential for leukemic growth. Coupling pooled CRISPR perturbations with single-cell RNA sequencing (Perturb-seq) further resolved the perturbation-induced alterations in regulatory networks, cell cycle states, and cellular hierarchies in primary AML samples. Together, these studies establish a general and robust framework for leveraging CRISPR-based functional genomics to directly dissect cancer dependencies and cellular heterogeneity in primary AML patient samples.

## In brief

Direct CRISPR functional genomics in primary patient samples holds promise for therapeutic target validation and discovery. Cao et al. develop an optimized CRISPR platform for genetic screening in primary acute myeloid leukemia samples. Integrated knockout, knockdown, and Perturb-seq screens dissect cancer vulnerabilities and cellular heterogeneity directly in patient-derived cells.

## Graphical Abstract

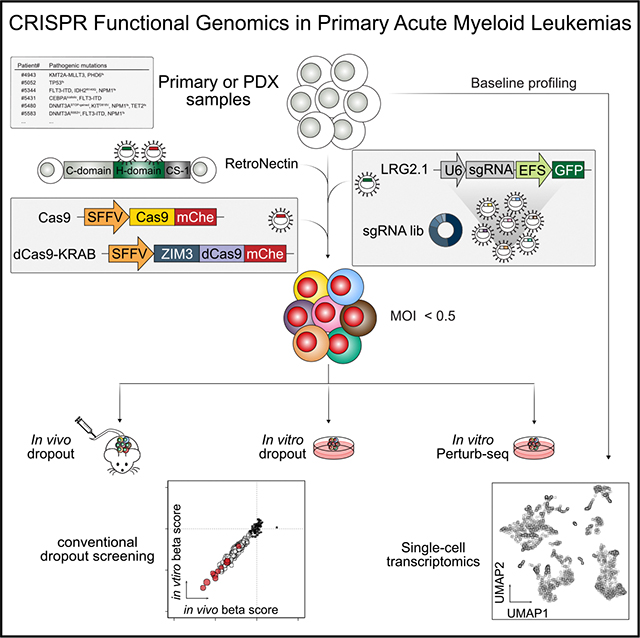

## INTRODUCTION

Acute myeloid leukemia (AML) is an aggressive and complex hematopoietic malignancy characterized by the uncontrolled proliferation of immature myeloid progenitor cells. Single-cell profiling studies have revealed significant inter- and intra-tumoral heterogeneity in AML, offering valuable insights into clonal diversity and disease progression. ^1–4^ At the molecular level, the key regulators of gene expression are frequently mutated or dysregulated through genetic and epigenetic mechanisms. ^5–8^ Traditionally, cancer research has largely relied on genetically engineered mouse models and human cancer cell lines harboring specific oncogenic driver mutations to identify cancer dependencies and explore therapeutic targets. Although these models have enabled important discoveries, the persistently high relapse rate in AML underscores their limitations. ^9^ This challenge stems partly from our incomplete understanding of the gene regulatory networks that support leukemia survival within heterogeneous subpopulations, as well as the inability of preclinical models to fully recapitulate the complex mutational landscapes observed in individual patients. While CRISPR functional genetic screens have facilitated the large-scale identification of leukemia-specific vulnerabilities, ^10–13^ most targets have been discovered and validated in cell lines or mouse models that incompletely reflect primary disease. Collectively, these limitations highlight the unmet need to develop functional genomic strategies that directly leverage patient primary samples for high-throughput genetic studies in AML.

Recent efforts have been made to optimize CRISPR-based knockout genetic screening in patient-derived xenograft (PDX) cells of AML. In these studies, AML PDX cells were lentivirally transduced with the Cas9 gene and subsequently expanded either through a short-term *in vitro* culture in cytokine cocktails or via orthotopic transplantation into immunocompromised mice prior to conventional “dropout” genetic screens. ^14–16^ However, this approach is both time-consuming and labor-intensive, and it is applicable only to a subset of primary patient samples that are amenable to *in vivo* expansion in immunodeficient mice. Moreover, conventional dropout screens primarily identify essential genes for growth-related phenotypes, thereby failing to capture complex transcriptional changes and to dissect cellular hierarchies within individual tumors, which is a critical limitation given the pronounced intra- and inter-patient heterogeneity observed in AML. Integrated CRISPR and single-cell approaches, including perturbations with single-cell RNA sequencing (Perturb-seq), CRISP-seq, and CROP-seq, ^17–20^ enable pooled perturbations with single-cell transcriptomic readouts but have been largely restricted to cancer cell lines or mouse models. To our knowledge, there have been no reports of their successful implementation in PDX or primary AML patient samples, likely associated with delivering and establishing CRISPR systems in patient-derived cells with high gene editing efficiency.

In this study, we developed an enhanced CRISPR functional genomic platform to investigate cancer vulnerabilities in primary AML patient samples spanning diverse pathogenetic mutations. Among the 22 primary patient samples tested, ∼86% (19 of 22) supported single-gene perturbation, and ∼73% (16 of 22) were suitable for pooled sgRNA library screening. We performed both *in vitro* and *in vivo* CRISPR knockout and CRISPR interference (CRISPRi) knockdown dropout genetic screens in patient-derived AML cells, including samples lacking established human cell line models. We also developed a Perturb-seq pipeline targeting 39 known AML-related therapeutic genes in patient samples, revealing distinct regulatory networks in supporting AML. From targeted screens encompassing 39 AML-related genes and 197 chromatin regulatory domains, we identified differential dependencies on chromatin regulators among patient samples. Collectively, this study establishes a general framework for leveraging CRISPR functional genomics to dissect cancer vulnerabilities and cellular heterogeneity in primary AML patient samples.

## DESIGN

CRISPR functional genomics in primary tumor samples offers a powerful opportunity to evaluate therapeutic efficacy and uncover cancer dependencies or resistance mechanisms. However, direct CRISPR genetic screening in AML primary cells remains technically challenging due to low lentiviral delivery efficiency, the need for Cas9 pre-installation, and the requirements for *in vivo* cell expansion. To overcome these limitations, we performed a series of optimizations. First, to enhance the CRISPR gene editing efficiency, lentiviral vectors were optimized to increase Cas9 and sgRNA expression while incorporating fluorescent markers to enable tracking of CRISPR-positive populations without antibiotic selection. Second, to improve transduction efficiency and compatibility with limited cell numbers, delivery methods were tailored for AML patient-derived cells using ultracentrifuged-concentrated virus and biological bridge molecules to enhance virus-cell interactions. These optimizations enabled robust CRISPR-based genetic studies across AML patient samples with diverse mutational landscapes. We established an experimental framework supporting both CRISPR knockout and CRISPRi knockdown modalities, enabling interrogation of coding genes and *cis-*regulatory elements directly in primary AML samples.

## RESULTS

### Optimization of a robust CRISPR knockout system in AML PDX and primary patient cells

**Figure 1.**
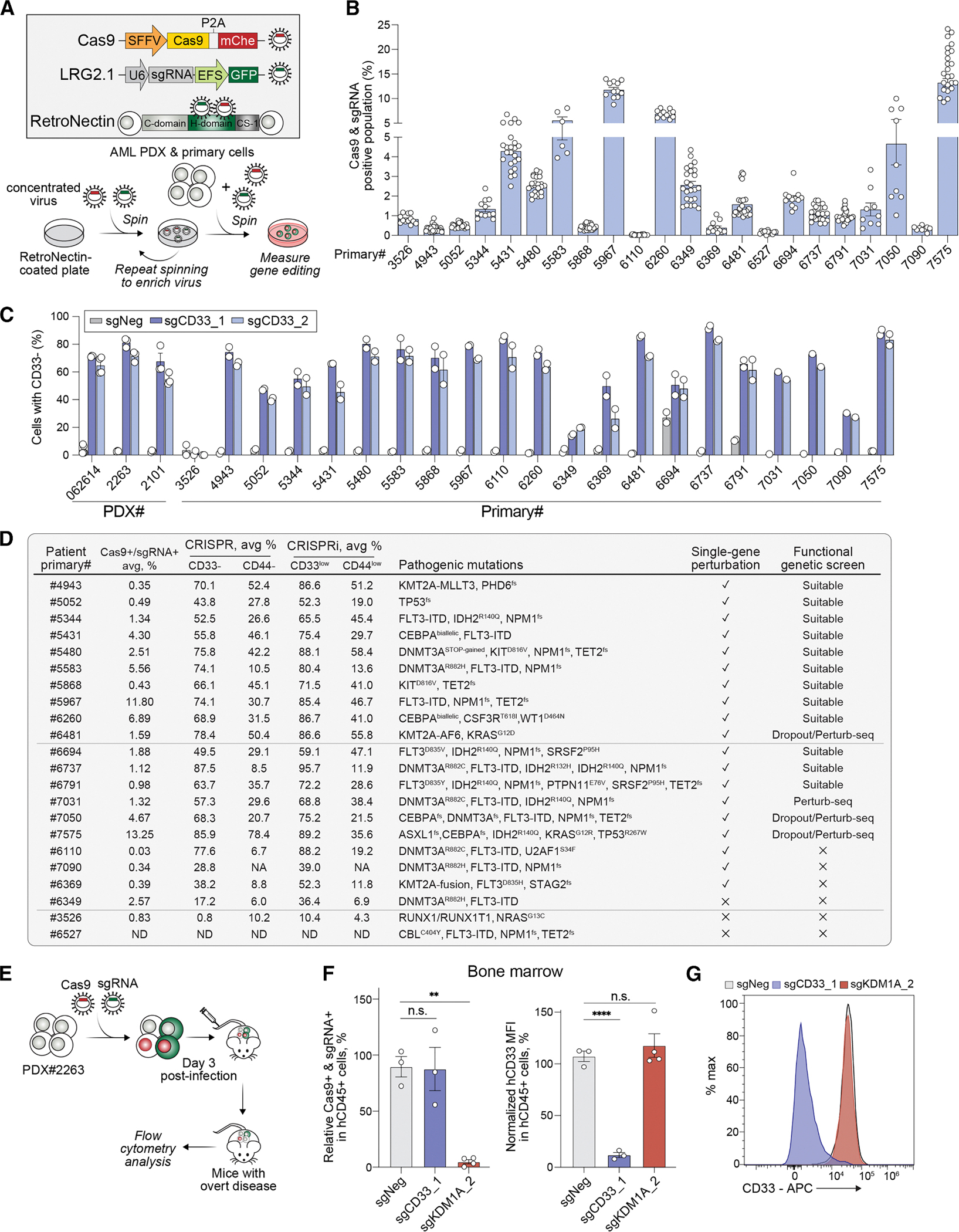
Establishment of a robust CRISPR knockout system in AML PDX and primary patient cells (A) Configuration of optimized CRISPR vectors used in PDX and primary AML cells (top). mChe, mCherry fluorescence marker. C-domain and CS-1 domain, cell-binding domain. H-domain, heparin-binding domain. Experimental workflow of virus transduction in PDX and primary AML cells (bottom). (B) Quantification of Cas9 and sgRNA double-positive percentage in a panel of 22 primary AML samples on day 5 post-transduction (*n* = 6–12). (C) Quantification of CRISPR-mediated CD33 perturbation efficiency at day 7 post-transduction with indicated sgRNAs, measured by flow cytometry (*n* = 3–5 for PDX samples, *n* = 2 for primary AML cells). (D) Summary of information of primary AML cells used in this paper, including sample number, average Cas9+/sgRNA+ double-positive percentage, average CD33 and CD44 perturbation efficiency by CRISPR and CRISPRi, pathogenic mutations, and suitability for single-gene perturbation or functional genetic screen. (E) Schematic of *in vivo* CRISPR competition assays. PDX#2263 cells were lentivirally transduced with the indicated sgRNA and Cas9, generating mixed populations that were transplanted into immunodeficient mice via tail vein injection. In overt leukemia, human leukemia cells (CD45^+^) from bone marrow and spleen were isolated and analyzed by flow cytometry analysis. (F) Relative abundance of Cas9+/sgRNA+ (mChe+/GFP+) cells in hCD45+ bone marrow cells at leukemia onset, normalized to day 3 post-transduction (*n* = 3–4) (left), and CD33 expression in Cas9+/sgRNA+ hCD45+ cells (*n* = 3–4) (right). MFI, mean fluorescence intensity. *p* values were calculated via unpaired two-tailed *t* test. ** *p* < 0.01, **** *p* < 0.0001; n.s., not significant. (G) Representative flow cytometry of CD33 expression following indicated CRISPR perturbation in Cas9+/sgRNA+ hCD45+ bone marrow cells. All error bars represent the mean ± SEM.

To enable functional genetic screening in human AML patient samples, including both PDX and primary specimens, we optimized a lentiviral CRISPR genome editing system in which stable viral integration provides an intrinsic barcode for high-throughput functional assays. We first employed dual lentiviral vectors expressing either Cas9 or sgRNA linked to fluorescent markers instead of antibiotic resistance genes, minimizing perturbations to cell state and sample heterogeneity. The fluorescent markers allowed us to track the double Cas9 and sgRNA positive population and assess both lentiviral transduction and gene editing efficiencies (Figure 1A). To evaluate system performance, we targeted non-essential genes such as the cell surface markers CD33 and CD44 in AML primary samples *in vitro*. To improve lentiviral transduction efficiency, we concentrated the virus-containing supernatant by ultracentrifugation and applied the concentrate to RetroNectin-coated plates. RetroNectin, a recombinant fragment of fibronectin, bridges lentiviral particles to integrin receptors on hematopoietic cells, increasing the local virus concentration at the cell surface and enhancing viral entry. ^21^ Finally, incorporation of a stronger SFFV promoter and an improved sgRNA backbone (LRG2.1) ^22^ further increased both transduction and gene editing efficiencies (Figure S1A). In comparison to conventional polybrene-based viral delivery and electroporation-based CRISPR ribonucleoprotein delivery methods, our optimized lentiviral system achieved effective gene editing with significantly lower cellular toxicity in both AML PDX and primary cells (Figure S1B).

Using this system, we performed CD33 and CD44 knockout experiments in 3 AML PDX samples and 22 primary AML patient samples with diverse mutational backgrounds, including high-risk genotypes or those lacking representative human cell line models, such as *IDH1/IDH2* mutations, complex *TP53* mutants, ^23^ and *DNMT3A*, *FLT3-ITD*, and *NPM1* triple mutations ^7^ (Table S1). Double-positive population transduction efficiencies ranged from 0.03% to 13.25% (Figure 1B), with knockout efficiencies of 17.2%–87.5% for CD33 and 6.0%–78.4% for CD44 (Figures 1C and S1C–S1E). Overall, ∼86% (19/22) of primary patient samples supported single-gene perturbation experiments (defined as >20% gene editing efficiency for CD33 on average), and ∼73% (16/22) were compatible with pooled sgRNA library screening (defined as >0.3% transduction efficiency and >40% gene editing efficiency for CD33 on average) (Figure 1D).

We next evaluated the performance of our optimized CRISPR system *in vivo*. Using PDX#2263 cells harboring a *KMT2A*::*AFF1* (*MLL-AF4*) translocation, we performed orthotopic transplantation and targeted either the cell surface marker CD33 or the histone demethylase *KDM1A* (also known as *LSD1*), a known AML therapeutic target. ^24^ Cells were simultaneously transduced with Cas9 and the respective sgRNA, generating four populations: non-transduced, Cas9 only, sgRNA only, and double positive (Cas9 and sgRNA). These mixed populations were intravenously injected into immunodeficient mice, and flow cytometry was performed ∼3 weeks post injection when overt disease was observed (Figure 1E). We observed that the double-positive Cas9+/sgKDM1A+ population was depleted ∼20-fold relative to pre-transplant levels (Figures 1F and S1F). Similarly, human leukemic cells isolated from both bone marrow and spleen exhibited an 84%–89% reduction in CD33 expression in the double-positive Cas9⁺/sgCD33⁺ population (Figures 1F, 1G, S1G, and S1H). Collectively, these results demonstrate the robustness of our engineered CRISPR system for direct genetic perturbation in both AML PDX and primary patient samples.

### CRISPR knockout genetic screens in AML PDX and primary patient samples reveal shared and distinct dependencies

**Figure 2.**
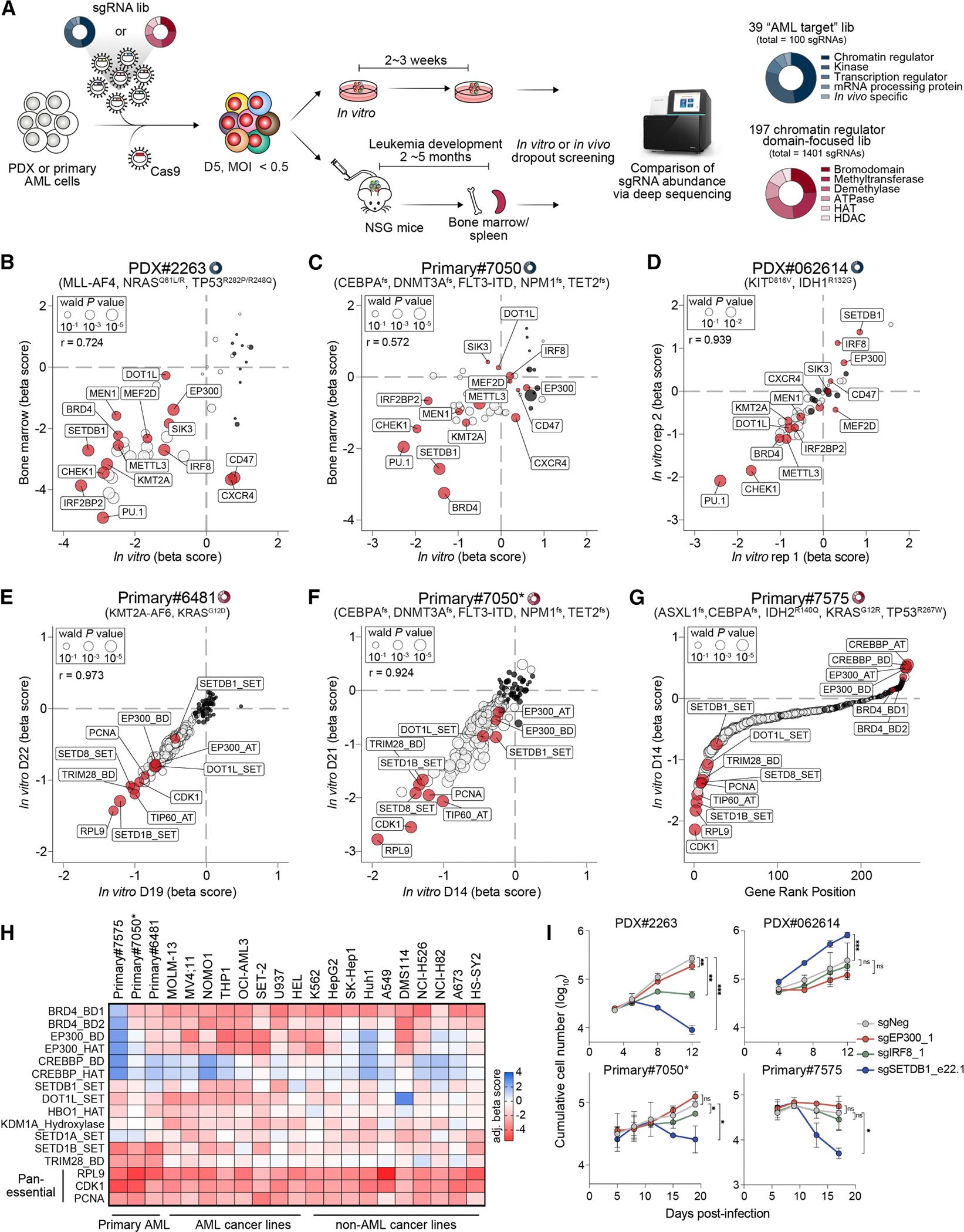
CRISPR knockout dropout screens in AML PDX and primary patient samples revealed shared and distinct dependencies (A) Schematic workflow of *in vitro* and *in vivo* CRISPR screens in PDX and primary AML cells using the 39 “AML target” library or the 197 domain-focused chromatin regulator library. Primary cells were cultured with myeloid cytokines and lentivirally transduced with transduction of the pooled sgRNA library and Cas9. One portion of cells was maintained *in vitro* for 2–3 weeks, during which double-positive cells, GFP+/mCherry+, were sorted for dropout screen. In parallel, cells were intravenously transplanted into immunodeficient mice, and double-positive human leukemic cells (hCD45+) and GFP+/mCherry+ were isolated from bone marrow and spleen at overt leukemic for *in vivo* dropout screen. (B–D) Scatterplots showing correlations of target gene beta scores from CRISPR screens using the 39 “AML target” library between (B) *in vitro* and bone marrow in PDX#2263 cells, (C) *in vitro* and bone marrow in Primary#7050 cells, and (D) two independent *in vitro* replicates of PDX#062614 cells. For *in vivo* screens, median beta scores from 3 independent replicates were shown. (E and F) Scatterplots showing correlations of target gene beta scores from CRISPR screens using a 197 chromatin regulator library between two independent *in vitro* replicates of (E) Primary#6481 cells and (F) Primary#7050* cells. Primary#7050* cells were isolated from peripheral blood one day after the initial isolation of Primary#7050 cells from the same patient. (G) Scatterplots showing ranked target gene beta scores from the 197 chromatin regulator library screen in Primary#7575 cells *in vitro*. In (B)–(G), black circles represent negative control sgRNAs, while red circles denote shared and distinct gene dependencies as described in the text. Circle size corresponds to –log10(Wald *p* value) from the MAGeCK-MLE module. (H) Heatmap summarizing results from the 197 chromatin regulator domain-focused CRISPR screens across primary AML cells, AML cell lines, and other non-AML cancer cell lines. (I) Proliferation assays of indicated sgRNAs in Cas9-expressing PDX#2263 (upper left), PDX#062614 (upper right), Primary#7050* (lower left), and Primary#7575 (lower right) cells. Plots depict cumulative sgRNA+/Cas9+ cell numbers over time (*n* = 3). All error bars indicate geometric mean with 95% confidence interval (CI). *p* values were calculated via two-way ANOVA followed by a multiple comparisons test. * *p* < 0.05, ** *p* < 0.01, *** *p* < 0.001; ns, not significant.

After establishing efficient CRISPR gene editing in primary AML patient samples, we evaluated its performance for pooled dropout genetic screening *in vitro* and *in vivo*. We first designed a small-scale sgRNA library targeting 39 known AML dependencies, including chromatin regulators, transcription factors, mRNA processing proteins, and kinases (Figure 2A; Table S2). ^11,13,25–42^ To enable *in vivo* screening, sgRNAs were curated from previous studies and targeted annotated functional domains to maximize knockout efficiency.^43^ Library quality was confirmed by *in vitro* dropout screens in the MOLM-13 AML cells, where known dependencies were re-identified with expected dropout strengths (Figure S2A). We next performed parallel *in vitro* and *in vivo* screens in PDX#2263 cells (harboring *KMT2A::AFF1 (MLL-AF4)*, *NRAS*, and *TP53* mutations) and Primary#7050 (harboring mutations in *CEBPA*, *DNMT3A*, *FLT3-ITD*, *NPM1*, and *TET2*; Figures 2A–2C). AML cells were cultured with myeloid cytokines and lentivirally transduced with both Cas9 and the sgRNA library at a low multiplicity of infection (MOI < 0.5). Cells were split for *in vitro* culture or orthotopic transplantation into immunodeficient mice. Cas9 and sgRNA double-positive populations, with at least 400-fold coverage of the sgRNA library, were collected at defined time points: day 4 post-transduction as an input reference, 2–3 weeks post-transduction for *in vitro* screening, and at the onset of overt leukemic symptoms from bone marrow and spleen for *in vivo* screening (Figure 2A). sgRNA abundance was quantified by deep sequencing and analyzed using the MAGeCK pipeline ^44^ to calculate gene essentiality beta scores.

Examination of the screening results revealed a high correlation between *in vitro* and *in vivo* screening results, as well as between bone marrow and spleen materials isolated from independent *in vivo* experiments (Figures 2B, 2C, and S2B–S2D). Similar correlations were observed in *in vitro* screens of PDX#062614 (harboring mutations in *IDH1* and *KIT*) (Figure 2D), indicating that *in vitro* dropout genetic screening could potentially serve as a proxy for identifying cell-autonomous factors required for leukemia cell proliferation and survival. Consistent with their known roles, pan-leukemia-essential genes such as *PU.1*, *CHEK1*, and *BRD4* were strongly depleted in all 3 patient samples in both *in vitro* and *in vivo* screens. AML subtype-specific dependencies, such as *KMT2A*, *DOT1L*, and *MEN1*, showed varying degrees of depletion among the 3 patient samples. Additionally, lineage-specific or leukemia-type-specific dependencies were observed. For example, the transcription factors *IRF8* and *MEF2D*, which are downstream targets of *KMT2A* rearrangement, and *SIK3*, which regulates the activity of *MEF2C*, a key of *KMT2A* rearrangement, were preferentially depleted in PDX#2263, which harbors a *KMT2A* translocation (Figures 2B–2D). ^4,13,28,45,46^

To evaluate the target-discovery potential of CRISPR functional genomics in patient samples, we performed *in vitro* dropout genetic screens using a larger, domain-focused chromatin regulator sgRNA library. This library comprised 1,401 sgRNAs, including 5–6 sgRNAs for each of 197 chromatin-associated domains, as well as both positive and negative controls ^13^ (Table S3). 3 primary patient samples with distinct chromatin or transcriptional mutations were selected: Primary#6481 harbors a *KMT2A::AFDN (MLL-AF6)* translocation, Primary#7050 carries mutations in *CEBPA*, *DNMT3A*, *NPM1*, and *TET2*, and Primary#7575 harbors mutations in *ASXL1*, *CEBPA*, *IDH2*, and *TP53*. Robust depletion of control sgRNAs targeting pan-essential genes confirmed screen performance (Figures 2E–2G). High concordance between shared sgRNAs in the 39-AML gene and 197-chromatin domain libraries in Primary#7050 supported reproducibility (Figure S2E). These screens revealed variable dependencies on individual chromatin regulators (Figures 2E–2G). A cross-analysis of previous dropout screens using the same sgRNA library showed that sgRNAs targeting *SETD1B* and *TRIM28* were preferentially depleted in these 3 primary AML samples compared with various leukemia and solid tumor cancer cell lines (Figure 2H).

Comparison of screening data from both the 39 AML target library and the 197 chromatin regulator domain-focused library across 6 PDX and primary patient samples revealed an opposing requirement for *BRD4*, *EP300,* and *SETDB1* in supporting leukemic cell proliferation (Figures 2B–2G). Although all three genes are generally considered essential for AML survival in a broad collection of AML cell lines,^26 ,37,42,47^ DepMap genome-wide CRISPR screen data confirmed their general requirement in AML cells with various oncogenic drivers (Figure S2F). In our screens, sgRNAs targeting the histone acetyltransferase *EP300* were significantly depleted in PDX#2263 and Primary#6481 (Figures 2B and 2E), both harboring *KMT2A* translocations, aligning with previous reports indicating that *EP300* perturbation impairs oncogenic transcriptional programs in multiple AML subtypes. ^48^ Conversely, sgRNAs targeting either the HAT or BD domains of EP300 were enriched in PDX#062614 and Primary#7575, which harbor *IDH1* or *IDH2* mutations, respectively (Figures 2D and 2G), suggesting that EP300 inhibition may cooperate with the oncometabolite 2-hydroxyglutarate pathway in supporting *IDH*-mutant AML proliferation. ^49^ *SETDB1* sgRNAs were depleted in PDX#2263 and Primary#7050 (and modestly in Primary#7575) but enriched in PDX#062614 (Figures 2B–2D and 2G). SETDB1 catalyzes histone H3 lysine 9 trimethylation (H3K9me3) to repress endogenous retrovirus (ERV) expression and interferon-stimulated genes in AML, and genetic depletion of *SETDB1* or pharmacological inhibition of H3K9 methyltransferase led to growth defects in a broad range of AML. ^26,50–52^ Validation using individual sgRNAs targeting *EP300*, *IRF8*, and *SETDB1* confirmed these dependencies in both PDX and primary samples, consistent with the results from the genetic screens (Figures 2I and S2G). For example, cell proliferation assays, measuring cumulative growth over time, and colony formation assays confirmed the requirement for *SETDB1* in PDX#2263, Primary#7050, and Primary#7575, whereas *SETDB1* perturbation accelerated cell proliferation in PDX#062614 (Figures 2I and S2G). These results indicate that different driver mutations in the transcriptional machinery may confer distinct dependencies on specific chromatin regulators, underscoring the utility of CRISPR functional genomics in primary patient samples for both therapeutic target validation and identification.

### CRISPRi knockdown genetic screens identify functional *cis-*regulatory elements in AML PDXs

**Figure 3.**
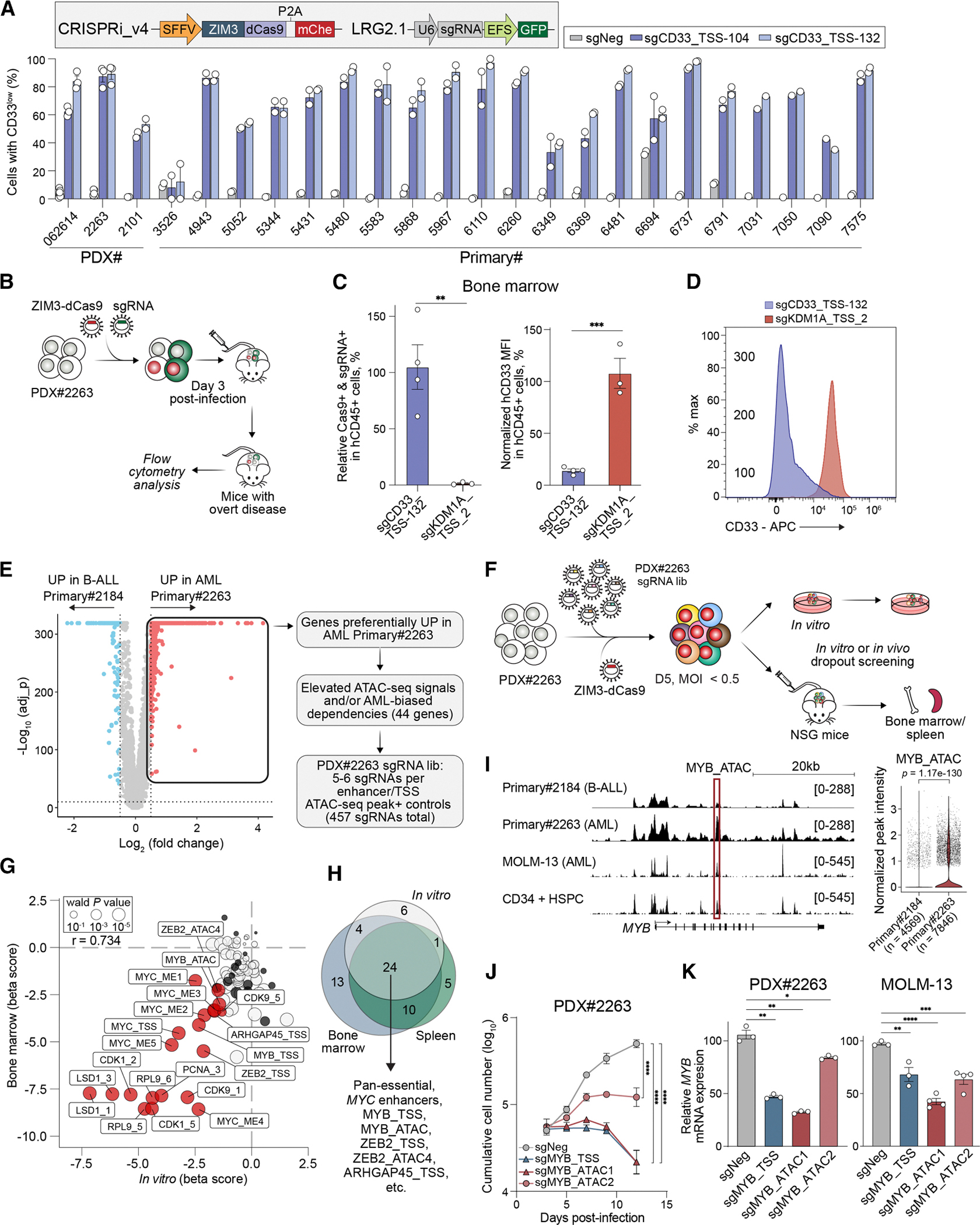
CRISPRi knockdown genetic screens identified functional *cis-*regulatory elements in AML PDXs (A) Configuration of the optimized CRISPRi vectors used in PDX and primary AML cells (top). Quantification of CRISPRi-mediated CD33 knockdown at day 7 post-transduction with indicated sgRNAs in a panel of AML patient-derived samples, measured by flow cytometry (*n* = 3–6 for PDX#062614 and PDX#2263, *n* = 2 for PDX#2101 and primary AML cells). (B) Schematic of *in vivo* CRISPRi competition assays. PDX#2263 cells were lentivirally transduced with indicated sgRNA and ZIM3-dCas9, followed by transplantation into immunodeficient mice via tail vein injection. In overt leukemia, human CD45 ^+^ (hCD45+) cells from bone marrow and spleen were isolated for flow cytometry analysis. (C) Percentage of dCas9-ZIM3+/sgRNA+ (mChe+/GFP+) cells among hCD45+ bone marrow cells at leukemia onset, normalized to day 3 post-transduction (*n* = 3–4) (left). *p* values were calculated via unpaired two-tailed *t* test. ** *p* < 0.01, *** *p* < 0.001. All error bars represent the mean ± SEM. (D) Representative flow cytometry of CD33 expression following CRISPRi perturbation in dCas9-ZIM3+/sgRNA+/hCD45+ bone marrow cells. (E) Volcano plot of scRNA-seq data comparing lineage-switch myeloid blasts (Primary#2263, relapsed post-CD19 CAR-T therapy) and pre-switch B-ALL blasts (Primary#2184). ^55^ Red and blue dots indicate significantly upregulated or downregulated genes in Primary#2263 (|log2FC| > 0.5 and *p*adj < 0.01), respectively (left). Schematic of the custom sgRNA library targeting Primary#2263-specific *cis-*elements (right). (F) Workflow of CRISPRi screens in PDX#2263 cells. Cells transduced with a pooled sgRNA library and ZIM3-dCas9 were cultured *in vitro* or transplanted *in vivo*. Double-positive/GFP+/mCherry+ hCD45+ cells were sorted, and sgRNAs were PCR-amplified for deep sequencing. (G) Correlations of target beta scores between *in vitro* and bone marrow CRISPRi screens. Black circles represent negative control sgRNAs, while red circles denote known dependencies as described in the text. Circle size corresponds to –log10(Wald *p* value) from the MAGeCK-MLE module. (H) Venn diagram comparing dependencies (beta score < − 1) identified by *in vitro* and *in vivo* screens. (I) MYB_ATAC activity across samples. Left: ATAC-seq tracks at the MYB locus, ^56^ highlighting the MYB_ATAC region (chr6:135,194,929–135,196,024; hg38). Right: violin plot of MYB_ATAC accessibility in Primary#2184 (merged from 4,569 single cells) and Primary#2263 (merged 7,846 single cells). (J) Proliferation assays of indicated sgRNAs in ZIM3-dCas9-expressing PDX#2263 cells, plotted as cumulative sgRNA+/ZIM3-dCas9+ cell number over time. (*n* = 3.) Error bars represent the geometric mean with 95% CI. (K) RT-qPCR analysis of MYB mRNA expression in ZIM3-dCas9+ PDX#2263 (left) and MOLM-13 (right) cells transduced with indicated sgRNAs (*n* = 3). *p* values were calculated via one-way ANOVA followed by a multiple comparisons test. * *p* < 0.05, ** *p* < 0.01, *** *p* < 0.001, **** *p* < 0.0001. Unless otherwise specified, data are presented as mean ± SD.

In addition to CRISPR-mediated gene knockout, CRISPRi offers a valuable approach to evaluate gene dosage effects and to investigate *cis-*regulatory DNA elements that support cancer cell survival. Despite CRISPRi having been widely applied in model systems, ^53^ it has not yet been implemented in AML PDX or primary patient samples. To expand the toolkit, we optimized a CRISPRi system for AML PDX and primary samples by building on our lentiviral CRISPR knockout framework (Figure 1A). In PDX#062614 cells, replacing the commonly used ZNF10 KRAB domain with the ZIM3 KRAB domain ^54^ enhanced gene repression efficiency (Figure S3A). Our optimized CRISPRi system achieved silencing of CD33, with reductions in expression ranging from 36.4% to 95.7%, and of CD44, with reductions from 6.9% to 58.4%, in 3 AML PDX samples and 22 primary patient samples (Figures 3A, 1D, and S3B–S3D). *In vivo* validation in PDX#2263 further showed a ∼74-fold depletion of the Cas9+/sgKDM1A+ population relative to single-positive and non-transduced cells (Figures 3B, 3C, and S3E). Additionally, human leukemic cells isolated from bone marrow and spleen exhibited a ∼66%–85% reduction in CD33 expression in the Cas9+/sgCD33+ population compared with pre-transplantation levels (Figures 3D, S3F, and S3G). These results demonstrate that our optimized CRISPRi system is effective for gene silencing and target validation in AML PDX and primary patient samples.

To evaluate the *in vivo* performance of our optimized CRISPRi system and its potential to uncover leukemia-essential *cis-*regulatory elements, we selected the PDX#2263 model. This PDX was derived from a patient (Primary#2184) initially diagnosed with B cell acute lymphoblastic leukemia, who received B-lineage-directed therapy and later relapsed with AML through a lineage switch mechanism. ^55^ We focused on identifying *de novo cis-*regulatory elements that emerged during this lineage transition and evaluating their functional relevance to PDX#2263 survival. A comparison of scRNA-seq data from relapsed AML blasts (Primary#2263) used to generate PDX#2263 and the original B-ALL blasts (Primary#2184) identified 742 genes significantly upregulated in Primary#2263 (log_2_ FC > 0.5, *p*adj < 0.01) (Figure 3E). From this list, we selected 44 candidates based on AML-biased dependencies in the DepMap dataset and/or elevated chromatin accessibility in ATAC-seq profiles. We designed a pooled CRISPRi sgRNA library targeting putative enhancers and promoters of these genes, with each regulatory element targeted by 5–6 sgRNAs. The library also included positive controls targeting known AML enhancers (e.g., *MYC*) and promoters of pan-essential genes (Figure 3E). PDX#2263 cells were lentivirally transduced with this library and subjected to parallel *in vitro* and *in vivo* dropout screens using the same workflow as the CRISPR knockout experiments (Figures 3F and 2A).

In both *in vitro* and *in vivo* CRISPRi screens of PDX#2263, all positive control sgRNAs were strongly depleted, confirming the robustness of the optimized CRISPRi system (Figures 3G and S3H). Targeting the known *MYC* enhancer modules (ME1–ME5)^57,58^ revealed that all 5 were required for PDX#2263 proliferation, with ME4 and ME5 targeting sgRNAs exhibiting the strongest depletion (Figures 3G and S3H). The screens also nominated the *ARHGAP45* as a dependency (Figures 3G, 3H, and S3H), which we subsequently validated as a selective vulnerability in *ARHGAP45*-high hematopoietic malignancies, including AML, in a recent study. ^16^ Additionally, we identified uncharacterized *cis-*elements within the intronic regions of leukemia-essential transcription factors (labeled MYB_ATAC and ZEB2_ATAC; Figures 3G and 3H). While the *MYB* promoter remained constitutively open throughout hematopoiesis and in malignancies, the MYB_ATAC region showed dynamic accessibility during disease progression. In normal hematopoiesis, MYB_ATAC was open in myeloid progenitors, including lymphoid-primed multipotent progenitors (LMPP), common myeloid progenitors (CMP), and granulocyte–macrophage progenitors (GMP), but closed in lymphoid and mature myeloid cells (Figure S3I). Of note, MYB_ATAC accessibility and *MYB* expression were increased in Primary#2263 AML cells relative to the original B-ALL sample (Primary#2184) (Figure 3I). MYB_ATAC accessibility further increased during adult AML development and was associated with H3K27ac enrichment in pediatric and adult AML but not in diagnostic or relapsed B-ALL patient samples (Figures S3J–S3L), suggesting AML-specific activation of this element to maintain aberrant *MYB* expression. To validate our screening results and assess the role of the MYB_ATAC in regulating *MYB* expression, we transduced PDX#2263 cells with ZIM3-dCas9 and two independent sgRNAs targeting this *cis-*regulatory element. Both sgRNAs reduced cell growth in PDX#2263 cells compared with the negative control, as shown by proliferation assays (Figure 3J). Reverse-transcription quantitative PCR (RT-qPCR) analysis confirmed that targeting MYB_ATAC decreased *MYB* expression in both PDX#2263 and MOLM-13 cells (Figure 3K). Collectively, these findings underscore the feasibility and target-discovery potential of a high-throughput CRISPRi platform for dissecting *cis-*regulatory elements in patient-derived leukemia cells.

### Perturb-seq of AML patient cells reveal regulators of genes related to leukemia maintenance

**Figure 4.**
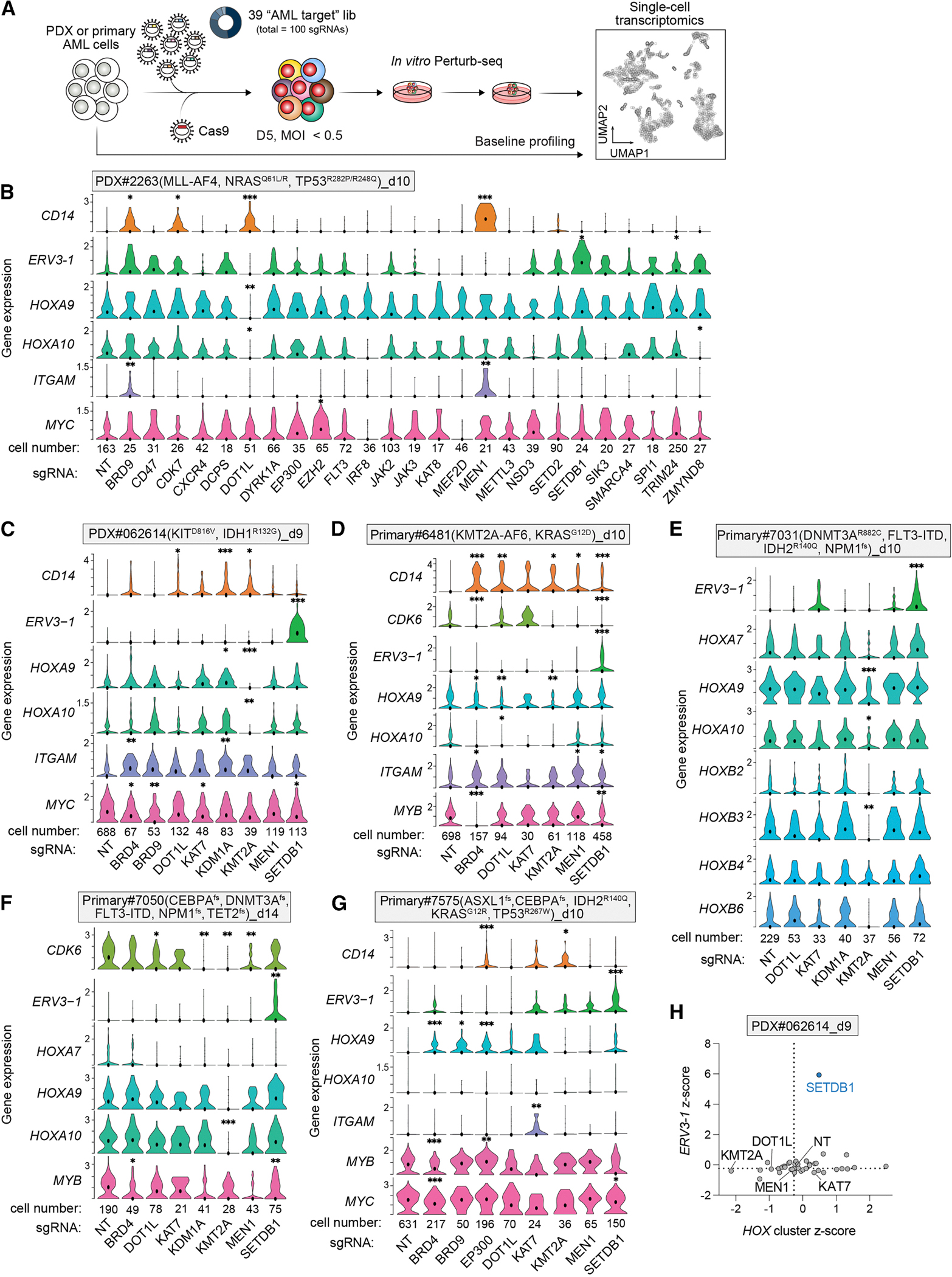
Perturb-seq of AML patient cells revealed regulators of genes related to leukemia maintenance (A) Schematic workflow of *in vitro* Perturb-seq in PDX and primary AML cells using the 39 AML target library. A small fraction of primary cells was subjected to scRNA-seq after DNase I treatment to serve as a baseline reference. Remaining cells were cultured with myeloid cytokines and lentivirally transduced with the pooled sgRNA library at low MOI (<0.5) along with Cas9. Cells were maintained *in vitro* for a short period (6–11 days), after which Cas9+/sgRNA+ double-positive cells were sorted for scRNA-seq and Perturb-seq analysis. (B–G) Violin plots showing expression of indicated genes (rows) following genetic perturbations (columns) across patient samples. (B) PDX#2263 at day 10 post-transduction. (C) PDX#062614 at day 9 post-transduction. (D) Primary#6481 at day 10 post-transduction. (E) Primary#7031 at day 10 post-transduction. (F) Primary#7050 at day 14 post-transduction. (G) Primary#7575 at day 10 post-transduction. NT, non-targeting control. (H) Perturbation effects of indicated sgRNAs on *HOX* cluster expression (*x* axis) and *ERV3–1* expression (*y* axis) in PDX#062614 at day 9 post-transduction. Effects are shown as z-statistics, with cell numbers indicated for each target. *p* values were calculated via the Wilcoxon rank-sum test followed by multiple hypothesis test correction. * *p* < 0.05, ** *p* < 0.01, *** *p* < 0.001.

CRISPR-based screens rely on comparing sgRNA library representation to evaluate phenotypes such as proliferation, cell death, or specific gene expression. However, performing individual screens for each phenotype is challenging with primary patient samples because of limited cell numbers and restricted expansion capacity. Next, we explored Perturb-seq, ^17,19,20^ which integrates CRISPR perturbations with scRNA-seq to capture early transcriptional changes before overt phenotypes emerge. We modified our CRISPR screening workflow (Figure 4A) by first generating baseline scRNA-seq profiles of AML PDX and primary patient samples, followed by Perturb-seq using the 39 AML target library in 6 patient samples with diverse mutation profiles (PDX#2263, PDX#062614, Primary#6481, Primary#7031, Primary#7050, and Primary#7575). Across these experiments, we detected a median of 3,078–21,768 unique molecular identifiers (UMIs) and 1,446–4,932 genes per cell, with 41.1%–68% of cells in Perturb-seq uniquely assigned an sgRNA (Figures S4A and S4B). Moreover, the number of cells associated with an individual sgRNA correlated with its essentiality beta score from conventional dropout screens (Figures S4C and S4D), suggesting that Perturb-seq cell counts can approximate growth effects. Uniform manifold approximation and projection (UMAP) of the scRNA-seq and Perturb-seq data (Figures S5A and S5B) showed that samples clustered primarily by patient ID rather than by experimental time point, suggesting that inter-patient transcriptional heterogeneity was the primary source of variation. Within individual patient samples, Perturb-seq profiles diverged from baseline, reflecting both differential expansion of heterogeneous subpopulations *in vitro* and CRISPR perturbation effects. An exception was the non-malignant lymphoid cells (including T, natural killer [NK], and B cells), which were clustered together regardless of patient IDs.

Next, we examined leukemia-related gene expression changes in the Perturb-seq data (Figures 4B–4H and S5C–S5E), beginning on the *HOX* gene cluster, which is dysregulated in ∼50% of AML cases.^59^ In 4 out of 6 patient samples, sgRNAs targeting *KMT2A* (oncogenic fusion or wild-type allele) significantly reduced expression of at least two *HOXA* genes, except in *TP53*-mutant Primary#7575, which lacked detectable *HOXA* expression, and PDX#2263, where *KMT2A* sgRNAs had low cell numbers (Figures 4B–4G). Additionally, sgRNAs targeting *MEN1*, which encodes the KMT2A-interaction protein MENIN, reduced *HOXA* cluster expression in the *DNMT3A/NPM1/FLT3-ITD*-mutant Primary#7050 (Figure 4F), in line with findings from a MENIN inhibitor study in *NPM1c/FLT3-ITD* cell lines.^60^ Because AML is characterized by impaired myeloid differentiation, we next examined markers of macrophage/monocyte differentiation such as CD11b (encoded by *ITGAM*) and CD14. In PDX#2263 cells, sgRNAs targeting *BRD9*, *KDM1A*, *PRMT5*, *DOT1L*, and components of the KMT2A complex strongly induced CD11b or CD14 expression (Figure 4B), consistent with previous studies using small-molecule inhibitors in *KMT2A*-rearranged AML.^24 ,29,61,62^ Similarly, in PDX#062614 cells, sgRNAs targeting *BRD4*, *KDM1A*, *DOT1L*, and KMT2A complex components induced CD14 expression (Figure 4C). Analysis of the DepMap dataset further supported that AML cell lines with high *HOXA9* expression were more dependent on *KMT2A* compared with those with low *HOXA9* expression (Figure S5F).

Finally, we assessed the expression of leukemia-essential transcription factors, including *MYC*, *MYB*, and the *IRF8-MEF2D* autoregulatory circuit, in the Perturb-seq data. Consistent with previous reports that *BRD4*, *BRD9*, and *KMT2A* regulate *MYC* and *MYB*,^29 ,42,63,64^ their sgRNAs led to the downregulation of *MYC* and *MYB* (Figures 4B–4G and S5C). The data also validated the regulatory circuit between *IRF8-MEF2D* and the chromatin reader *ZMYND8*^13 ,28,45^ (Figures 4B and S5C). This circuit was undetectable in the baseline scRNA-seq profiles of Primary#7050 and PDX#062614 (Figure S5D), consistent with the lack of depletion of *IRF8* or *MEF2D* sgRNAs in the corresponding dropout screens (Figures 2C and 2D). We further examined ERVs, which are typically silenced by H3K9me3 and whose reactivation can trigger interferon signaling and cell death in AML.^26^ Among the 39 perturbation targets, *SETDB1* depletion induced the strongest expression of *ERV3–1* across all 6 patient samples, including PDX#062614 (Figures 4B–4H and S5E). Of note, *SETDB1*-targeting sgRNAs were depleted in PDX#2263, Primary#7050, and Primary#7575 but enriched in PDX#062614. Altogether, our results demonstrate the utility of using Perturb-seq to evaluate the transcriptional impact and cellular effects of perturbing leukemia-essential genes directly in patient-derived cells.

### Perturb-seq analysis of proliferation-associated gene signatures predict target essentiality in AML

**Figure 5.**
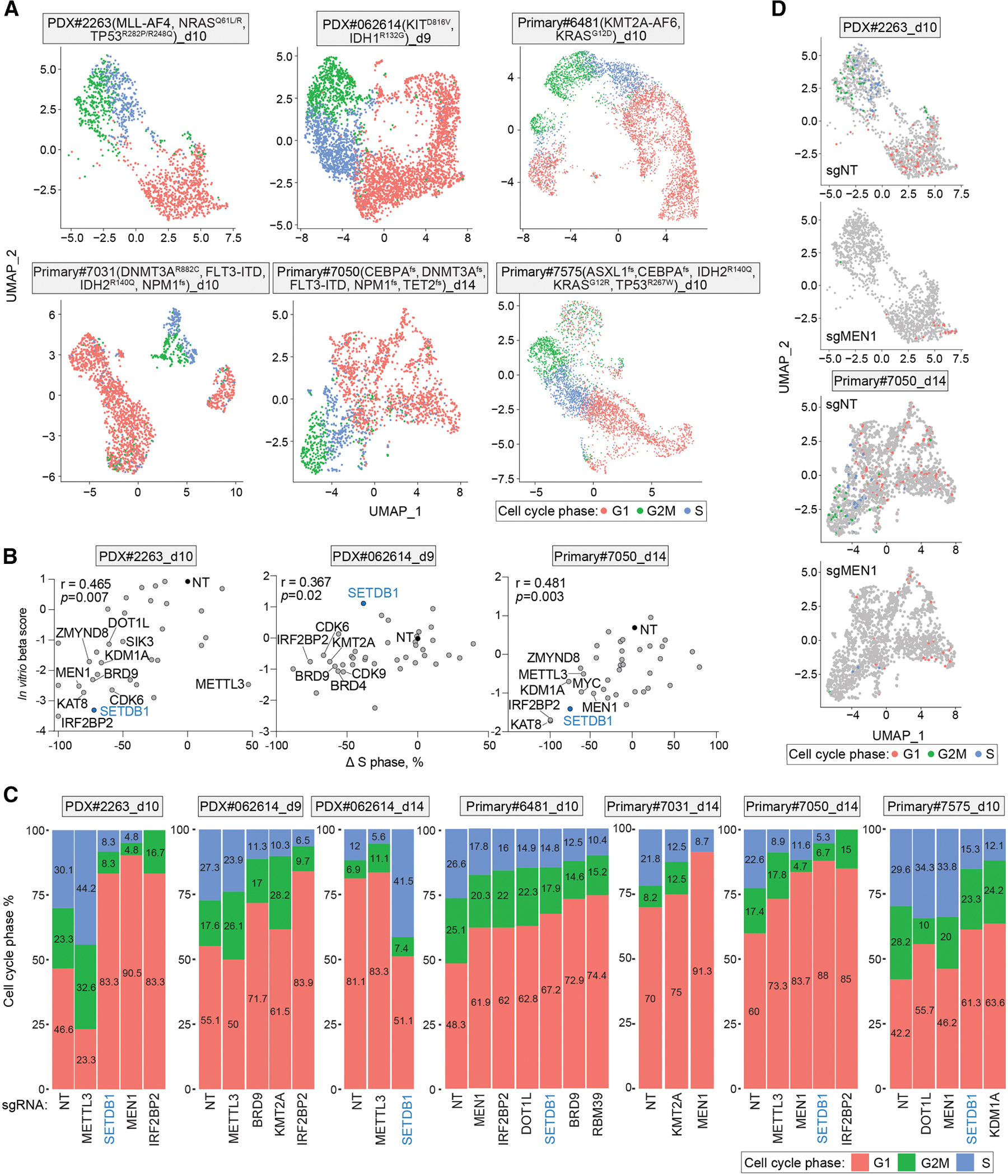
Perturb-seq analysis of proliferation-associated gene signatures predicted target essentiality in AML (A) UMAP visualization of cell cycle phases across indicated Perturb-seq samples. (B) Scatterplots showing correlations between the relative difference in S phase % and *in vitro* beta scores in PDX#2263 (day 10 post-transduction, left), PDX#062614 (day 9 post-transduction, middle), and Primary#7050 (day 14 post-transduction, right). Relative S-phase change was calculated as (S%_sgRNA − S%_NT)/S%_NT. NT, non-targeting control. (C) Bar graphs displaying proportions of cells in the S phase (top, blue), G2M phase (middle, green), and G1 phase (bottom, red) for the indicated sgRNAs across samples at indicated time points post-transduction: PDX#2263 (day 10), PDX#062614 (days 9 and 14), Primary#6481 (day 10), Primary#7031 (day 14), Primary#7050 (day 14), and Primary#7575 (day 10). Percentages for each phase are indicated. (D) UMAP plots highlighting cell cycle phases for individual cells transduced with either negative control sgNT (upper) or sgMEN1 (lower) in Perturb-seq data from PDX#2263 at day 10 post-transduction (upper) and Primary#7050 at day 14 post-transduction (lower).

In addition to inferring gene essentiality from Perturb-seq cell counts (Figures S4C and S4D), we investigated whether perturbation-induced transcriptional changes in cell cycle-regulated genes could predict gene essentiality in AML and potentially serve as an alternative to traditional dropout genetic screens. We hypothesized that changes in cell cycle composition could serve as a proxy for dependency. To test this, we used Mix-scape ^65^ to correct for cell cycle effects and applied UMAP to visualize sgRNA perturbations in each patient sample (Figures 5A, S6A, and S6B). Because some leukemia-essential sgRNAs caused strong cell depletion, analyses were restricted to sgRNAs with at least 10 mapped cells to ensure statistical robustness. Across 3 patient samples with matched *in vitro* dropout data, reductions in the fraction of S-phase cells relative to negative controls correlated with more negative essentiality beta scores (Figure 5B). For example, in 5 samples expressing *HOXA9* and *HOXA10*, sgRNAs targeting *KMT2A* or its partners (*MEN1* or *DOT1L*) reduced the fraction of S-phase cells, whereas no effect was observed in Primary#7575, which lacks *HOXA* expression (Figures 5C, 5D, and 4G). Furthermore, *SETDB1* perturbation strongly decreased the proportion of S-phase cells in PDX#2263, Primary#6481, Primary#7031, and Primary#7050, whereas in PDX#062614, an initial decrease in S-phase cells at day 9 was followed by an increase at day 14, consistent with the enrichment of *SETDB1* sgRNAs in the *in vitro* dropout screens (Figures 2D and 5C).

We then performed gene set enrichment analysis (GSEA) to further evaluate the impact of perturbations on leukemia-related gene signatures. We selected 38 representative gene sets that are strongly associated with AML biology and commonly used in AML studies: 11 associated positive regulators of cell growth (e.g., cell cycle, leukemia stem cell [LSC], and MYC-target gene sets), 14 linked to negative regulators of cell growth (e.g., myeloid differentiation, apoptosis, and inflammatory pathways), and 13 representing neutral or context-specific regulators (e.g., KMT2A-MLLT3 target genes and EZH2_module; Table S4). For each perturbation, we compared expression profiles to negative controls within the same Perturb-seq experiment and calculated normalized enrichment scores (NESs) across all gene sets. Principal component analysis revealed that growth-promoting and growth-inhibiting gene sets formed distinct clusters, while context-specific sets were more variable (Figure S6C). Consistently, NESs between MYC targets and the LSC signature were highly correlated, as were those between myeloid cell development and the TNF-α signaling pathway via nuclear factor κB (NF-κB), whereas positive and negative regulators were strongly anti-correlated (Figure S6D). Context-dependent gene sets, such as *MEF2C* or *KMT2A*-fusion signatures, were selectively downregulated following depletion of specific dependencies (e.g., *IRF8/MEF2D* or the KMT2A complex in PDX#2263) (Figure S6E), consistent with prior studies. ^13,28,30^ Perturbations of strong dependencies (e.g., KMT2A complex components in PDX#2263 and Primary#6481; *CDK7*, *KAT8*, *DOT1L*, and *IRF2BP2* in PDX#062614; and *MEN1*, *IRF2BP2*, and *KDM1A* in Primary#7050) tended to alter multiple leukemia-essential signatures (Figure S6F). Taken together, these results support that integrating cell counts, cell cycle analysis, and gene signature profiles from Perturb-seq provides a comprehensive approach to evaluating gene essentiality in AML.

### Perturb-seq projection onto normal hematopoietic trajectory uncover target gene impact on cellular composition

**Figure 6.**
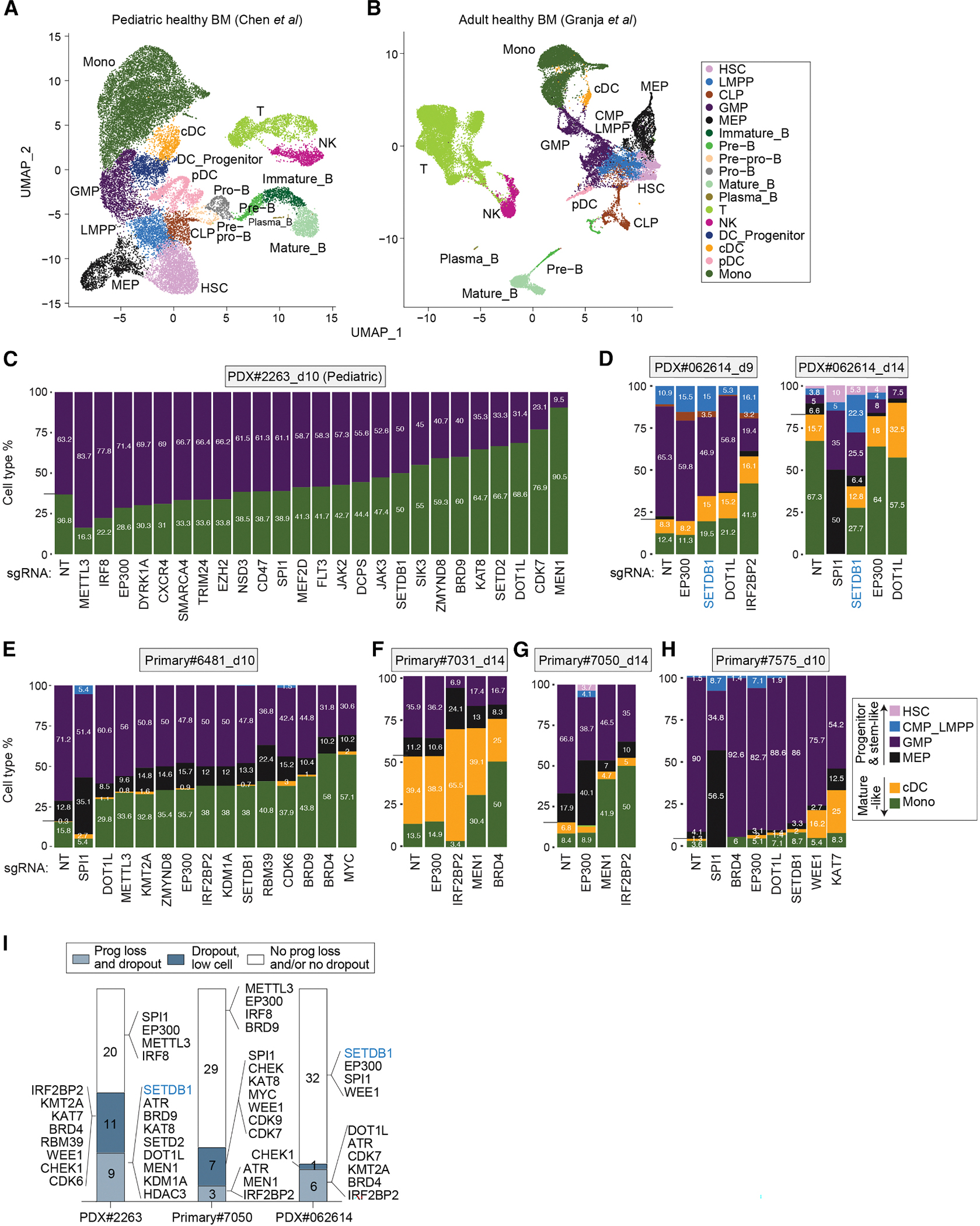
Perturb-seq projection onto normal hematopoietic trajectory revealed target gene impact on cellular composition (A) UMAP plots of bone marrow scRNA-seq data from healthy pediatric donors. Data retrieved from Chen et al. ^55^ (B) UMAP plots of bone marrow scRNA-seq from healthy adult donors. Data retrieved from Granja et al. ^67^ (C) Projection of Perturb-seq data from PDX#2263 at day 10 post-transduction with indicated sgRNA onto the healthy pediatric reference UMAP in (A). sgRNAs were ranked by the fraction of progenitor and stem cell population, with sgNT shown on the left. Only sgRNAs represented by ≥15 cells were included. Cell population percentages are indicated. (D–H) Representative projections of Perturb-seq data onto the adult reference UMAP in (B) from (D) PDX#062614 at days 9 and 14, (E) Primary#6481 at day 10, (F) Primary#7031 at day 14, (G) Primary#7050 at day 14, and (H) Primary#7575 at day 10 post-transduction with indicated sgRNAs. (I) Bar graphs depicting stratification of the 39-gene AML target library based on *in vitro* and *in vivo* CRISPR dropout screens, as well as *in vitro* Perturb-seq data in PDX#2263, Primary#7050, and PDX#062614 cells. Targets showing ≥20% progenitor cell loss and depletion in dropout screens (when available) are shown in the light blue, depleted targets with insufficient Perturb-seq cell numbers are shown in dark blue, and targets with no progenitor loss and/or no depletion are shown in white.

Single-cell analyses have revealed significant heterogeneity within AML tumors, with subpopulations exhibiting both progenitor-like and more differentiated mature cell states.^1 ,56,66^ We sought to assess the impact of individual genetic perturbations on leukemia cell state using our Perturb-seq dataset. We first reconstructed the UMAPs of pediatric and adult healthy bone marrow references using published datasets and methods^55 ,67^ and annotated hematopoietic populations based on established markers (Figures 6A and 6B). Because AML tumor evolution and cellular hierarchy mirror normal myeloid development,^1^ we projected scRNA-seq and Perturb-seq data onto the corresponding pediatric or adult references. Only perturbation targets represented by more than 15 cells in the scRNA-seq dataset were included and ranked based on the ratio of progenitor to mature cell types. Consistent with our prior findings,^55^ PDX#2263 myeloid blasts comprised both GMP (progenitor) and monocyte (mature)-like populations (Figures 6C and S7A). Other samples displayed greater heterogeneity, with 4–6 distinct cell states detected in Perturb-seq (Figures 6D–6H and S7B–S7D). The non-targeting control groups for Primary#7031 and PDX#062614 showed an increased proportion of mature cell populations at later time points, reflecting the limited and disproportionate expansion of specific subpopulations *in vitro* (Figures 6D, 6F, S7C, and S7D).

Stem and progenitor-like populations in AML are associated with poor clinical outcomes and resistance to cytotoxic chemotherapy ^68,69^ and targeted therapeutics. ^70,71^ We ranked 39 targets from primary patient screens based on their impact on depleting these populations. By integrating Perturb-seq with *in vitro* and *in vivo* CRISPR dropout screens, we classified the targets into three groups: (1) those reducing progenitor cells by at least 20% in Perturb-seq with beta scores < − 0.5 in pooled screens, (2) those with beta scores < − 0.5 and with less than 15 cell number counts in Perturb-seq, and (3) those that did not reduce progenitors in the Perturb-seq or were not depleted in either dropout screen (Figure 6I). Targets in groups (1) and (2) were considered potential therapeutic targets, as they could deplete both progenitor and blast populations.

This classification nominated *IRF2BP2*, *ATR*, *DOT1L*, and KMT2A complex components as effective targets in PDX#2263, PDX#062614, and Primary#7050, despite distinct mutational backgrounds (Figures 2B–2D, 6C, 6D, 6G, 6I, S7A, S7B, and S7D). In *KMT2A*-rearranged samples (PDX#2263 and Primary#6481), perturbation of *DOT1L*, *SETDB1*, *KMT2A*, or *MEN1* consistently reduced progenitor and stem-like cell populations (Figures 6C and 6E). In Primary#7031 and Primary#7050 (both harboring *DNMT3A/NPM1/FLT3-ITD* triple mutants), sgRNAs targeting *IRF2BP2*, *MEN1*, *BRD4*, *KMT2A*, and *CDK7* depleted progenitor and stem-like cells (Figures 6F–6H and S7C), suggesting that small-molecule inhibitors of these factors could be effective therapies for these genetically defined AML subtypes, for which no established human cell line models currently exist. In the *TP53*-mutant sample Primary#7575, progenitor-like populations were dominant, with 90% GMP and 4.1% MEP (megakaryocyte-erythroid progenitor), consistent with the aggressiveness of *TP53*-mutant AML.^72 ,73^ In this sample, most perturbations, except for *KAT7* inhibition, had little impact on reducing progenitor-like populations (Figure 6H), suggesting that targeting KAT7 may be effective in *TP53*-mutant AML.

Dependency nomination was generally consistent across genetic screens: sgRNAs that reduced progenitor cell fractions in Perturb-seq were also depleted in both *in vitro* and *in vivo* dropout screens (Figures 6I and S4D). However, several exceptions highlight the complementary information captured by each method. First, although sgRNAs targeting *METTL3*, an enzyme that catalyzes m6A methylation and destabilizes mRNA, were depleted in the dropout screens, they increased the proportion of progenitor and S-phase cells in PDX#2263 (Figures 2B, 5B, and 6C). This discrepancy is consistent with prior work showing that *METTL3* depletion in *KMT2A*-rearranged AML elevates the mRNA levels of *MYC* and *BCL2* but reduces their translation, ultimately suppressing leukemia cell proliferation, ^38^ an effect not fully captured by transcription-based approaches. Second, sgRNAs targeting *SPI1*, a lineage-specific AML dependency, were depleted in dropout screens and increased progenitor fractions in Perturb-seq (Figures 6D, 6E, 6H, and S7D), suggesting that a subset of *SPI1*-deficient cells temporarily adopts a progenitor-like transcriptional state before failing to expand, which aligns with *SPI1*’s role in hematopoietic lineage commitment and its established importance in AML.^74^ Third, *EP300* perturbation increased progenitor-like populations (e.g., HSC- and CMP/LMPP-like cells) in PDX#062614, Primary#7050, and Primary#7575 (Figures 6D, 6G–6H, 7E, and S7B). Of note, sgRNAs targeting distinct *EP300* domains were among the most enriched in the dropout screen of Primary#7575 (Figure 2E), suggesting that progenitor population expansion may drive positive selection of *EP300*-deficient cells. Collectively, these findings demonstrate that Perturb-seq offers valuable insights into the effects of individual genetic perturbations on cell state and subpopulation dynamics in patient-derived samples.

## DISCUSSION

Functional genomics studies in genetically engineered mouse models and established human cancer cell lines have identified numerous cancer dependencies and elucidated key molecular pathways in AML. ^10–13^ However, these systems often do not fully capture the diverse mutational landscapes and complex cellular heterogeneity inherent in primary tumors. Primary AML samples encompass a broad spectrum of pathogenic mutations and differentiation states,^2^ features often underrepresented in conventional models. In addressing this gap, we developed a robust CRISPR functional genomics platform optimized for primary AML samples. We validated the system through single-gene perturbation experiments in 3 PDX and 22 primary patient samples with varied mutational profiles, including those associated with poor prognosis and lacking representative human cell line models, and achieved successful genome editing in ∼88% (22 of 25) of the samples. As a proof-of-concept, we performed multiple *in vitro* and *in vivo* genetic screens to identify AML vulnerabilities directly in patient-derived cells. Together, these results establish a scalable platform that expands functional genomic studies to genetically diverse patient cohorts.

To facilitate high-throughput genetic studies, we developed an improved lentivirus-based CRISPR workflow that, unlike electroporation-based strategies, minimized cell death and maintained robust editing in primary AML cells (Figure S1B). Lentiviral integration also supports deep-sequencing-based analyses of functional phenotypes. We implemented 3 key optimizations: (1) enhanced Cas9 and sgRNA expression vectors, (2) an ultra-concentrated virus production protocol, and (3) the use of RetroNectin to enhance viral delivery by bridging viral particles to hematopoietic cells. Because RetroNectin engages integrins commonly expressed on hematopoietic cells (such as VLA-4 and VLA-5), this approach is potentially generalizable beyond AML, and further engineering may extend its applicability to additional primary cell types.

Using this system, we compared matched *in vitro* and *in vivo* dropout screens and observed strong concordance, underscoring the utility of *in vitro* assays for probing cell-autonomous dependencies. While *in vivo* experiments can capture non-cell-autonomous interactions within the tumor microenvironment, they require 2–5 months and are often limited by variable engraftment success. ^75^ By contrast, *in vitro* screens are completed within 2–3 weeks, a significant advantage given the limited expansion capacity and cell numbers available from primary patients. Moreover, the costs and labor associated with *in vivo* expansion further constrain its scalability. Since the *in vitro* system reliably recapitulates the cell-autonomous AML dependencies, it offers a rapid, cost-effective, and scalable approach for evaluating therapeutic targets and prioritizing patient-specific vulnerabilities.

Finally, the integrated CRISPR dropout and Perturb-seq screening platform enables systematic identification of cancer dependencies, including those unique to patients with complex mutations lacking representative cell lines. For example, screens in Primary#7050 and Primary#7031, both carrying clinically aggressive *DNMT3A*, *FLT3-ITD*, and *NPM1* triple mutations, revealed that *MEN1* and *IRF2BP2* perturbation reduced bulk tumor cells and suppressed immature progenitors (Figures 2C, 6F, and 6G). In Primary#7575, a rare but highly aggressive AML subtype with *KRAS*, *IDH2*, and *TP53* mutations (<2% of patients), >95% of cells exhibited progenitor-like states. Dropout screens showed little impact from most chromatin regulator disruptions, and even known AML dependencies like *EP300* and *BRD4* were positively selected. Our observation of *BRD4* sgRNA enrichment in Primary#7575 underscores the therapeutic resistance found in some patient subtypes. ^76,77^ Similarly, in PDX#062614, sgRNAs targeting *SETDB1* were positively selected in dropout screens, and single-cell analysis revealed the expansion of progenitor-like cells (Figures 2D and 6D), challenging the assumption of its universal essentiality in AML. Overall, these findings underscore the importance of validating therapeutic targets in clinically relevant samples and establish CRISPR functional genomics as a powerful framework for dissecting patient-specific vulnerabilities and advancing precision medicine in AML.

### Limitations of the study

Primary AML specimens frequently comprise multiple subclones, each harboring distinct combinations of pathogenic mutations. ^6,7^ While conventional dropout screens estimate gene essentiality across bulk populations and Perturb-seq provides transcriptional snapshots of perturbation-induced effects on cellular hierarchies, neither approach directly links functional dependencies to specific pathogenic genotypes within subclonal populations. As a result, the current system cannot resolve whether a given dependency is shared across all malignant clones or confined to a mutation-defined subpopulation. Moreover, while Perturb-seq is well suited for capturing transcriptional consequences of genetic perturbations, it may incompletely reflect regulatory effects occurring at the post-transcriptional or translational level. For example, although *METTL3* sgRNAs were significantly depleted in dropout screens (Figures 2B and 2D), Perturb-seq showed expansion of S-phase and progenitor cells, suggesting a proliferative advantage (Figures 5C and 6C). Prior studies have shown that *METTL3* loss in *KMT2A*-rearranged AML stabilizes *MYC* and *BCL2* transcripts but reduces their translation, ultimately impairing cell proliferation. ^25,38^ This discrepancy underscores the need for integrated, multi-layered analyses that combine fitness-based screening with transcriptional profiling. Finally, the scale of CRISPR functional screening in primary AML is inherently constrained by the low lentiviral transduction efficiency, typically in the single-digit range for freshly isolated patient cells even with our improved platform (Figure 1C). Together with restricted cell availability, this constraint currently limits the application of genome-wide pooled CRISPR libraries in primary AML.

## RESOURCE AVAILABILITY

### Lead contact

Further information and requests for resources and reagents should be directed to and will be fulfilled by the lead contact, Junwei Shi (jushi@upenn.edu).

### Materials availability

All plasmids will be deposited at Addgene and made available for public request (Addgene accession numbers are listed in the key resources table). PDX#2263 and PDX#2101 are available through the Pediatric High-Risk Cancer Preclinical Model Resource (FDA contract no. 75F40121C00137).

### Data and code availability

- scRNA-seq and Perturb-seq data have been deposited to GEO under accession number GSE221578 and are publicly available as of the date of publication. All other sequencing data were provided in Table S6. Published datasets analyzed in this study are listed in the key resources table.
- Analysis scripts used in this study are available on request or at https://github.com/tanlabcode/KMT2Ar_Paper ^55^ or https://github.com/GreenleafLab/MPAL-Single-Cell-2019, ^67^ and archived through Zenodo (10.5281/zenodo.18176272). Other codes are publicly available and cited in the key resources table.
- Any additional information required to reanalyze the data reported in this paper is available from the lead contact upon request.

## STAR★METHODS

### EXPERIMENTAL MODEL AND STUDY PARTICIPANT DETAILS

#### Cell line and patient-derived cell culture

All cell lines, or JCRB. MOLM-13 cells were cultured in RPMI-1640 (Gibco) supplemented with 10% Bovine Calf Serum (FCS) and 1% Penicillin/Streptomycin (P/S). HEK293T was cultured in DMEM (Corning) supplemented with 10% FCS and 1% P/S.

The primary AML and PDX samples were obtained from the Stem Cell and Xenograft Core Facility at The Perelman School of Medicine at the University of Pennsylvania or the Center for Childhood Cancer Research Biorepository at the Children’s Hospital of Philadelphia, with informed consent in accordance with the Declaration of Helsinki. Protocols used for this study were approved by the institutional review boards of the University of Pennsylvania and the Children’s Hospital of Philadelphia. The primary AML samples were frozen in FCS and 10% DMSO in liquid nitrogen for long-term storage. Patient clinical information is included in Table S1. For short-term *in vitro* maintenance, cells were cultured in BM media (IMDM supplemented with 15% Fetal Bovine Serum (FBS), 1% P/S, 1% GlutaMAX) and human cytokines, including 1:1000 dilution of the10ng/μl human IL-3 (Peprotech#200–03), 10ng/μl human FLT3 (Peprotech#300–19), 100ng/μl human SCF (Peprotech#300–07), 20ng/μl human IL-6 (Peprotech#200–06), and 10 ng/μl human TPO (Peprotech#300–18) stock.

#### Animals

Around 6–8-week-old Female NOD.Cg-Prkdc^scid^Il2rg^tm1Wjl^/SzJ (NSG) mice (Jax 005557) and NOD.Cg-Prkdc^scid^Il2rg^tm1Wjl^Tg (CMV-IL3, CSF2, KITLG) 1Eav/MloySzJ (NSGS) were purchased from the Jackson Laboratory. Mice were housed in an ASBL2 SPF barrier facility in group houses (maximum 5 animals/cage) and acclimatized for up to one week prior to tail-vein injection. All animal protocols were approved by the Institutional Animal Care and Use Committee (IACUC) at the Children’s Hospital of Philadelphia.

### METHOD DETAILS

#### Plasmid construction

All human sgRNAs were cloned into the BsmbI-digested LRG2.1 vector (Addgene #108098) after annealing the sense and antisense oligos. The SFFV-Cas9–2A-mCherry vector was created by swapping the EFS promoter of the LentiV_Cas9_Puro vector (Addgene #108100) with the SFFV promoter and then replacing the Puro with mCherry using the In-Fusion cloning strategy (Takara). The CRISPRi_v2 vector (SFFV-KRAB-dCas9–2A-mCherry) was created by replacing the EFS of the dCas9-KRAB-mCherry vector (Addgene#163956) with SFFV. The KRAB W8E mutation for the CRISPRi_v3 vector (SFFV-KRAB(W8E)-dCas9–2A-mCherry) was introduced to the CRISPRi_v2 vector through PCR mutagenesis. The ZIM3 gene for the CRISPRi_v4 vector (SFFV-ZIM3-dCas9–2A-mCherry) was cloned from the pLX303-ZIM3-KRAB-dCas9 vector (Addgene #154472). The sgRNA sequences for individual sgRNA experiments are listed in Table S5.

#### Virus production and transduction

Around 90%−100% confluent HEK293T cells in 10-cm plates were co-transfected with 10 μg of plasmid DNA, lentiviral packaging plasmids pPAX2 (5 μg) and VSVG (7.5 μg), 1 ml OPTI-MEM and cationic polymer reagent Polyethylenimine (PEI) at the final concentration of 1mg/mL as previously described. ^4^ To produce lentivirus in 6-well plates, 5 μg of the DNA, 2.5 μg of VSVG, 3.5 μg pPAX2, 1 mg/ml PEI and 500 μl OPTI-MEM were used for each well. The HEK293T cells were cultured for 5–6 hours before aspiration of the supernatant and replenishment with fresh DMEM media with 10% FCS. The supernatant containing lentivirus was collected at 24, 48, and 72hr post-transfection and filtered using a 0.45 μm PVDF membrane (Millex^®^-HV). To increase the virus titer, virus-containing supernatant can be incubated with 10% PEG-8000 overnight, and the mixture was spun down at 2500xg for 30min at 4°C. The supernatant was completely removed, and ∼1ml PBS/ BM media without cytokines was used to resuspend the pellet. The pellet can be stored at −80 °C.

For viral transduction in cell lines, cells were mixed with virus-containing supernatant and 4 μl/mL polybrene (2mg/ml) and spin-infected at 650 x g for 25 minutes at room temperature. Media was replenished the next day. For PDX and primary cell transduction, Retronectin (Takara) was used to avoid the cytotoxic effect associated with polybrene, following the manufacturer’s instructions with minor modifications. Briefly, 12-well non-treated plates were coated with 1ml of 20ng/μl Retronectin for 2 hours at 37°C or overnight at 4°C. The Retronectin was collected, and the plates were blocked with 1 ml of 2% BSA for 30 minutes at room temperature. The BSA was aspirated, and the plates were washed with 1 ml of PBS or HBSS. The plates were then ready to use or could be stored at 4°C for up to a week. To boost the infection efficiency for large viral vectors (e.g., the one containing Cas9), 1 ml of virus-containing supernatant was first applied to the plates and spun down at 750 x g for 30 minutes to allow the virus to attach to the bottom. The supernatant was aspirated, and the above steps could be repeated multiple times to enrich the virus further. To transduce the PDX and primary cells, cells were mixed with the virus, adjusted FBS (15% final), a human cytokine cocktail (human IL-3, FLT3, SCF, IL-6, and TPO) described above, and a 1:500 dilution of LentiBlast Premium (LBPX1500, OZBiosciences, which also enhances hematopoietic cell transduction). The media was replenished the next day with BM media supplemented with cytokines.

#### Electroporation

Electroporation was carried out using the P3 Primary Cell 4D-Nucleofector^™^ X Kit (Lonza) following the manufacturer’s protocol. For each reaction, 2 μl of 61 μM Cas9 protein (IDT) was combined with 1.4 μl of 100 μM sgRNA (IDT) in 16.6 μl of PBS and incubated at room temperature for at least 15 minutes to allow ribonucleoprotein (RNP) complex formation. Approximately 1 × 10⁵ PDX or primary patient-derived cells were used per reaction. Cells were washed once with PBS and resuspended in 80 μl of P3 buffer supplemented with 1.2 μl of Alt-R^®^ Cas9 Electroporation Enhancer (IDT). The RNP complex was then added to the cell suspension, and electroporation was performed using the Lonza 4D-Nucleofector^™^ X Unit with the “human CD34+ HSPC” preset program.

#### Flow cytometry

Cells were washed with Bone Marrow Wash buffer (BMW, PBS with 2% FBS, 1% P/S) and stained with indicated antibodies (human CD45-APC (1:50, BD #555485), murine PC7-CD45 (1:500, BioLegend 103113), murine PC5.5-CD45 (1:500, BioLegend 103131), human APC/Cy7-CD33 (1:100, BioLegend 303442), human APC-CD33 (1:100, BioLegend 303407), human APC-CD44 (1:200, BioLegend 397506), human PC7-CD44 (1:200 BioLegend 397513)). Flow cytometry was performed on a CytoFLEX (Beckman), sorting was performed on a FACSAria II flow cytometer (BD Biosciences) or MoFlo Astrios (Beckman), and data were analyzed with Flowjo10.

#### Construction of sgRNA library

Libraries were generated in a similar fashion as previously described. ^4,43^ For the “39 AML-related therapeutic targets” library, well-validated sgRNAs were retrieved from our and others’ previous studies. ^11,13,25–42^ For the PDX#2263 cis-element library, genes upregulated (|log2FC| >0.5, p_adj<0.01) in PDX#2263 cells were first identified, and genes with elevated ATAC-seq chromatin accessibility or displaying AML-biased dependency based on the Depmap database were manually curated. Around 5–6 sgRNAs were designed using Benchling (https://www.benchling.com/) to target the TSS or enhancer regions marked with ATAC-seq peaks. sgRNAs with high predicted on-target scores were selected, while those containing “TTTT” sequences, which can cause early PolIII transcription termination, were removed. Additionally, sgRNAs targeting more than one perfectly matched region in the human genome were removed. Non-targeting sgRNAs and essential gene-targeting sgRNAs were also included as negative and positive controls, respectively. Pooled sgRNA oligos were synthesized on an array platform (Twist Bioscience), amplified, and cloned into the Bsmb1-digested LRG2.1 vector using the Gibson cloning method (NEB). The quality and representation of each sgRNA in the library were verified via combination of sanger sequencing and deep sequencing analysis (data not shown). The sgRNA library sequences and corresponding CRISPR dropout screening results are provided in Tables S2, S3, and S6.

#### *In vivo* CRISPR/CRISPRi validation experiments

To test the efficiency of *in vivo* CRISPR and CRISPRi, individual sgRNAs and Cas9 or ZIM3-dCas9 were simultaneously transduced into PDX#2263 cells after thawing and recovery for 24 hours. On day 3 post-infection, expression of GFP and mCherry reporters were measured using a CytoFLEX (Beckman). NSG mice were allowed to acclimatize for up to one week before tail-vein injection. Mice were treated with 25 mg of Busulfan (Meitheal, NDC 71288–116-11) per kg of mouse weight at least 24 hours prior to injection of CRISPR-transduced PDX#2263 cells. Approximately 8×10^4^ cells were transplanted through the tail vein of Busulfan-treated mice. Mice displayed overt leukemia-related symptoms 3–4 weeks post-transplantation and were subsequently euthanized for analysis of leukemia burden in BM and SP (indicated by hCD45+/mCD45-) and gene editing efficiency of CRISPR and CRISPRi.

#### Competition-based proliferation assay in patient cells

PDX or primary AML cells were lentivirally transduced with the indicated sgRNAs (co-expressed with a GFP reporter in the LRG2.1 vector) and Cas9 co-expressed with an mCherry reporter. To assess proliferative competition between Cas9+/sgRNA+ double-positive cells and wild-type populations, the proportion of GFP+/mCherry+ cells was monitored over time. Fluorescence was measured every 5 days starting from day 3 or day 4 post-infection until day 13 or 14 using a CytoFLEX (Beckman). The fold changes in double-positive population normalized to the first time point were used for analysis.

#### RT-qPCR

Total RNA was isolated using the Direct-zol RNA Miniprep Plus Kit (ZYMO) with the in-column DNAse I treatment following the manufacturer’s protocol and then reverse transcribed using qScript cDNA SuperMix. qPCR was performed with SYBR green PCR master mix on the ABI ViiA 7 Real-Time PCR System. All results were normalized to *GAPDH*. Primer sequences used for RT-qPCR are listed in Table S5.

#### *In vitro* and *In vivo* CRISPR screens

To determine the titer of the pooled sgRNA libraries for the PDX cell experiments, ∼5×10^5^ cells were transduced with a serial dilution of the virus. To ensure that individual cells received only a single copy of sgRNA during transduction, the multiplicity of infection (MOI) was set between 0.3–0.5, confirmed by measuring GFP% on day 3 post-infection using a CytoFLEX (Beckman). The pooled sgRNA library virus had a much lower titer in the primary patient cells compared to the PDX samples, and the MOI was lower than 0.3. Therefore, the maximum volume of the pooled sgRNA library virus was used for the screenings in primary patient cells.

*In vitro* and *in vivo* CRISPR screens were performed by transducing cells with indicated libraries, followed by transduction of Cas9 (for the “39 AML therapeutic-related target” library) or ZIM3-dCas9 (for the 2263-specific cis-element library) on the next day. On day 3 post-infection of the sgRNA library, the GFP% was measured by a CytoFLEX (Beckman). An aliquot of cells was kept in culture for the *in vitro* screening, and the remaining cells were washed with PBS to remove the culture media. Then, 200 μl of PBS-resuspended cells were injected into each sub-lethally irradiated (2.5 Gy) immunodeficient NSG mouse through tail vein injection (for the “39 AML therapeutic-related target” library screen in PDX#2263 cells, 8×10 ^5^ unsorted cells were transplanted into each NSG mouse; for the “39 AML therapeutic-related target” library screen in primary#7050 cells, 4–10 ×10^6^ unsorted cells were transplanted into each NSG mouse; for the 2263-specific cis-element library screen in PDX#2263 cells, 3×10^6^ unsorted cells were transplanted into each NSG mouse). At this time point, cells were not FACS sorted of sgRNA and Cas9 double positivity to prevent cell loss and stress during sorting. On day 4 post-infection of the sgRNA libraries, a portion of the cells in culture were saved as inputs representing > 1000x of the sgRNA libraries. For *in vitro* screens, cells were kept growing for around 14 days, maintained for at least 1000x sgRNA representation in mCherry+ cells, and collected at the end of the screens. For *in vivo* screens, mice were euthanized when displaying leukemia-related symptoms (for PDX#2263 cells was ∼3–4 weeks; for primary#7050 cells was ∼12 weeks). Lysis buffer (Roche) was used to lyse red blood cells on ice for 5 min, followed by hCD45/mCD45 antibody staining and analysis of leukemia burden using a CytoFLEX (Beckman). It was noted that leukemia burden in BM could be very high (>90%) for primary cells, even if mice only displayed mild leukemia-related symptoms; therefore, mice could be sacrificed earlier for *in vivo* screens as desired. Cells were then stored in 20% FCS and 10% DMSO in liquid nitrogen until use.

For sequencing library preparation, GFP+/mCherry+/hCD45+ cells were sorted by FACS on a FACSAria II flow cytometer (BD Biosciences) or MoFlo Astrios (Beckman). Genomic DNA extraction was performed using the Quick-DNA Miniprep Kit (ZYMO) following the manufacturer’s protocol. PCR amplification of integrated sgRNA cassettes was previously described ^81^ with minor modifications. Briefly, approximately 300 ng of DNA was PCR amplified with a custom stacking barcode to produce an approximately 50 ng of PCR amplicon. The PCR amplicon was size selected using AMPure XP beads (Beckman) or sparQ PureMag Beads (Quantabio/VWR) by applying 0.6x beads for the first round (with beads discarded), 0.4x beads for second-round selection (with supernatant discarded), and 2x wash with 80% ethanol. The PCR products were then dissolved in 30 μl of PCR water. Illumina-compatible sequencing adaptors were introduced to the barcode-embedded PCR products with 8 cycles of PCR amplification, followed by 1x sparQ PureMag Bead selection of the ∼320bp final products. The libraries were analyzed and quantified using the Qubit dsDNA HS assay kit (Thermo Fisher) and the Bioanalyzer DNA 1000 kit (Agilent). Finally, libraries with different barcodes were pooled and sequenced together on either a Miseq or Nextseq 500 sequencer with 75 bp single- or paired-end reads.

Sequencing libraries were demultiplexed, trimmed, and aligned to the reference sgRNA library as previously described. ^42^ The sequencing data with raw read counts for each sgRNA was further analyzed using MAGeCK-MLE as previously described. ^44,82^ A beta score (similar to log_2_ (Fold Change)) was calculated for each target and control by the MAGeCK-MLE model. To calculate beta scores for non-targeting negative controls, every 3–4 negative controls were pooled as a single target. The data was visualized using Prism 9. The sgRNA library sequences and corresponding CRISPR dropout screening results are provided in in Tables S2, S3, and S6.

#### scRNA-seq

To prepare the scRNA-seq library, primary cells were thawed in PBS+5%FBS, and treated with DNase I (AMPD1–1KT, Sigma) for 5 min at room temperature. The cells were then washed and resuspended with PBS+0.04% BSA. Viability was checked by Trypan Blue (Thermo), and if the viability was lower than 95%, live cells were purified using the Dead cell removal kit (MACS). Subsequently, scRNA-seq was performed on viable cells according to the Chromium Next GEM Single Cell 5^′^ v2 protocol. Libraries were quantified using the Qubit dsDNA HS assay kit (Thermo Fisher) and the High Sensitivity DNA Bioanalyzer Kit (Agilent) and analyzed by the NEBNext Library Quant Kit for Illumina. Finally, pair-end sequencing was conducted on a Nova-seq 6000 using a Read 1 26, i7 index 10, i5 index 10, Read 2 90bp format.

#### Perturb-seq

For *in vitro* Perturb-seq, CRISPR-transduced cells were maintained in tissue culture dish for 9–14 days. For *in vivo* Perturb-seq, leukemia cells were harvested from BM on day 12 post-transplant. GFP+/mCherry+ double positive leukemia cells were sorted, washed 3 times, resuspended in PBS+0.04% BSA, and checked for viability. Samples were subject to the Perturb-seq protocol adapted from ECCITE-seq ^83,84^ with small modifications, without antibody-hashtag labelling. Briefly, the cell suspension containing 20,000 live cells was spiked with 2μM sg_RT_v4 oligo (AGCAAGTGAGAAGCATCGTGTCAAAGCACCGACTCGGTGCCAC). The cells were barcoded using the 10xGenomics platform, following the Chromium Next GEM Single Cell 5’ v2 protocol. sgRNA was amplified together with cDNA using a spike-in Guide-tag_additive (AGCAAGTGAGAAGCATCGTGTC), and separated from cDNA by 0.6x SPRIselect beads (Beckman) (beads bound cDNA while supernatant contained sgRNAs). sgRNA was further amplified with primers incorporated with TotalSeq_A_Dual_Index, and purified by 1.2x SPRIselect beads (Beckman). cDNA was amplified and purified following the 10x Genomics protocol. Libraries were analyzed using the Qubit dsDNA HS assay kit (Thermo Fisher) and the High Sensitivity DNA Bioanalyzer Kit (Agilent), and quantified by NEBNext Library Quant Kit for Illumina. The sequencing libraries were pooled with a ratio consisting of 90% cDNA libraries and 10% sgRNA libraries, and pair-end sequenced on a Nova-seq 6000 using a Read 1 26, i7 index 10, i5 index 10, Read 2 90bp format.

#### scRNA-seq computational pipeline

The scRNA-seq computational pipeline was executed using R Studio running R (version 4.0.5). All scRNA-seq data from Perturb-seq and non-Perturb-seq experiments were first processed using 10x Genomics Cell Ranger (version 6.1.2) to generate the gene by cell count matrices. These count matrices were then loaded into Seurat (version 4.1.1)^85^ objects as an “RNA” assay. For each sample, we ran a standard Seurat pipeline where cells were filtered only to include those cells with greater than 100 genes, greater than 500 total UMIs total, and less than 10% of UMIs mapping to mitochondrial genes. For Perturb-seq data of PDX#2263 at day 10 post-infection of sgRNA and on day 15 post-infection of sgRNA, we filtered for greater than 2000 total UMIs because there were many low-quality/non-cells in the data. Then, we normalized the data using Seurat’s Normalize Data function with default parameters. We selected the top 2000 variable genes using Seurat’s FindVariableFeatures function with default parameters. We further filtered genes to only keep those that had non-zero expression in at least 1% of the cells in the sample. These filtered top variable genes were used as features in Seurat’s ScaleData function. The total number of UMIs and the % of UMIs mapping to mitochondrial genes were used as variables to regress against in the ScaleData function. All other parameters were set to their default values. Principal component analysis (PCA) was performed using Seurat’s RunPCA function with 50 principal components. To visualize the data, Uniform Manifold Approximation and Projection (UMAP) was performed using Seurat’s RunUMAP function with the top 50 principal components.

For all Perturb-seq samples, 10x Genomics Cell Ranger was also used to generate sgRNA by cell count matrices. These count matrices were loaded into the scRNA-seq Seurat objects as a “GDO” (guide-derived oligos) assay. For each Perturb-seq sample, we normalized the GDO data using Seurat’s NormalizeData function using centered log ratio (CLR) as the normalization method and normalizing over cells (margin=2). We then used Seurat’s MULTIseqDemux function, with autoThresh = TRUE, maxiter = 100, and qrange = seq(from = 0.01, to = 0.99, by = 0.01) to optimize parameters. This function assigns each cell as either 1) a unique guide, 2) as “Negative” (no guide), or 3) as “Doublet” (two guides). Cells with a maximum GDO count less than 5 were considered as “Negative” as well as cells assigned a unique guide that did not have the maximum GDO count in the cell. This step was performed so only the highest confidence single-guide-assigned-cells were used. Cells assigned “Negative” and “Doublet” were filtered out. Next, we used Mixscape’s CalcPerturbSig function ^65^ to calculate a perturbation signature using PCA with the top 40 dimensions and 20 nearest neighbors. This perturbation signature was stored as a new “PRTB” assay in the Seurat object. The PRTB data were scaled using the RNA assay’s variable features as features in Seurat’s ScaleData function, setting do.scale = FALSE, do.center = TRUE. Then, PCA was performed using Seurat’s RunPCA function. To visualize the data, UMAP was performed using Seurat’s RunUMAP function with the top 40 principal components.

#### Annotating scRNA-seq cell types using healthy references

We used two public scRNA-seq datasets derived from healthy donors to annotate cell types in our scRNA-seq data. We used the healthy reference from ^55^ to annotate samples derived from pediatric patients (i.e. ID 2263) and the healthy reference from ^67^ to annotate samples derived from adult patients (i.e. ID 7050, 7031, 062614). For the Chen et al. healthy reference, we used the same Seurat object as the original paper, however, RunUMAP was applied on the pre-existing PCs with parameters return.model=TRUE, dims=1:20, seed.use=42, n.neighbors=30, min.dist=0.5 to replicate the original UMAP to the best of our ability. This was done because the original Seurat object did not have the UMAP model stored, which is required for the cell type annotation. For the healthy reference from, ^67^ since the original data was shared as a SingleCellExperiment and did not have an associated UMAP model stored, we created a new Seurat object from the original gene by cell count matrix and ran a standard pipeline to reproduce the original SingleCellExperiment results to our best ability. Some similar cell types were combined. For each scRNA-seq sample, to find a set of anchors between the healthy reference and the sample, we used Seurat’s FindTransferAnchors function with reduction=“pcaproject”, dims=1:30, and reference.reduction=“pca”. Then, we used Seurat’s MapQuery function with the anchors found using FindTransferAnchors to annotate our samples.

#### Identification of differentially expressed genes

The differential expressed genes between B-ALL 2184 and AML 2263 were identified using the FindMarkers function in Seurat 4.0.5. An LR test was performed on the raw counts and the total UMI count per cell was regressed out as a confounding factor. Differentially expressed genes were defined as those with an average |log_2_ (fold_change)| > 0.5 and p_adj < 0.05.

The differential gene expression analysis comparing HSC v.s. others in PDX#062614_d14 sgSETDB1 was performed using Seurat’s FindMarkers with default parameters (two-sided Wilcoxon Rank Sum test, min.pct = 0.1, logfc.threshold = 0.25). The Benjamini-Hochberg false discovery rate (FDR) was used to correct for multiple hypothesis testing.

#### Gene set enrichment analysis (GSEA)

The average log_2_ fold change (log_2_ FC) between cells assigned to guides for a given CRISPR target gene and cells with non-targeting/control guides was computed using Seurat’s FindMarkers function with default parameters. These log_2_FC values were then ordered in decreasing order and passed to clusterProfiler’s GSEA function. The p-value adjustment value used was “FDR”. A custom list of 38 gene sets derived from previous studies listed in Table S4 ^86^ was used for the GSEA analysis. The GSEA function returned the normalized enrichment score (NES) and false discovery rate (FDR) for each gene set. For each CRISPR target gene, only gene sets with FDR < 0.25 were plotted.

#### Visualization of scATAC-Seq data

scATAC-Seq data of 2184 and AML 2263 were downloaded from Chen et al. Uniquely mapped reads from leukemic cells were used to compute a normalized signal for each 50 bp bin across the genome. The normalized signal was then uploaded and visualized in the UCSC genome browser: https://genome.ucsc.edu/.

### QUANTIFICATION AND STATISTICAL ANALYSIS

All error bars represent the mean ± standard error. For correlation analysis, Pearson’s R was used. Statistic methods to calculate *p* value were indicated in the figure or or annotated in the respective figure legends. *p* value was calculated using GraphPad Prism 9 or R.

## Supplementary Material

1

2

3

4

5

6

7

SUPPLEMENTAL INFORMATION

Supplemental information can be found online at https://doi.org/10.1016/j.molcel.2026.02.003.

## Figures and Tables

**KEY RESOURCES TABLE:** 

REAGENT or RESOURCE	SOURCE	IDENTIFIER
Antibodies		
PE/Cyanine7 anti-mouse CD45	Biolegend	Cat # 103113; RRID: AB_312978
APC Mouse Anti-Human CD45	BD Biosciences	Cat # 555485; RRID: AB_398600
PerCP/Cyanine5.5 anti-mouse CD45	Biolegend	Cat # 103131; RRID: AB_893344
human APC-CD33	Biolegend	Cat# 303407; RRID: AB_314351
human APC-CD44	BioLegend	Cat# 397506;RRID: AB_2814375
PE/Cyanine7 anti-human CD44	BioLegend	Cat# 397513; RRID: AB_2888761
Bacterial and virus strains		
MultiShot^™^ StripWell Stbl3^™^ Competent Cells	Invitrogen^™^	Cat #C739601
MegaX DH10B T1R Electrocomp^™^ Cells	Invitrogen^™^	Cat# C640003
Biological samples		
List of patients included in the study.	This study	Table S5
Chemicals, peptides, and recombinant proteins		
AMPure XP	Beckman Coulter	A63880
Penicillin/ Streptomycin	Thermo Fisher Scientific	15140122
Polyethylenimine, PEI	Polysciences, INC	23966
OPTI-MEM	Thermo Fisher Scientific	31985070
Hexadimethrine Bromide, Polybrene	Sigma-Aldrich	H9268
T4 polynucleotide kinase	New England Biolabs	M0201L
Agarose, Standard, Low Electroendosmosis (EEO)	Avantor	A426–07
Dimethyl Sulfoxide	Sigma-Aldrich	D2650
Human IL-3	Peprotech	Cat #200–03
Human FLT3L	Peprotech	Cat #300–19
Human SCF	Peprotech	Cat #300–07
Human IL-6	Peprotech	Cat #200–06
Human TPO	Peprotech	Cat #300–18
Lenti-X concentrator	Takara Bio	Cat # 631232
LentiBlast Premium	OZBiosciences	Cat # LBPX1500
Retronectin	Takara Bio	Cat # T100B
Alt-R^™^ S.p. Cas9 Nuclease V3	IDT	Cat # 1081058
Alt-R^®^ Cas9 Electroporation Enhancer	IDT	Cat # 1075915
BsmBI-v2	NEB	Cat # R0739S
Methylcellulose-based medium with recombinant cytokines for human cells	STEMCELL Technology	Cat # H4434
T4 DNA ligase	NEB	M0202L
T7 DNA ligase	NEB	M0318L
PEG-8000	Sigma-Aldrich	P2139
Busulfan	Meitheal	NDC 71288–116-11
sparQ PureMag Beads	Quantabio/VWR	95196–060
KAPA HiFi HotStart ReadyMix	Roche	07958935001
DNase I	Sigma-Aldrich	AMPD1–1KT
Critical commercial assays		
In-Fusion HD Cloning Kit	Takara Bio	638909
2x Phusion Master Mix	Thermo Scientific	F-548
Direct-zol RNA Miniprep Plus	Zymo Research	R2072
Agilent High Sensitivity DNA Kit	Agilent	5067–4626
QIAquick PCR Purification Kit	QIAGEN	28104
Quick-DNA Miniprep Kit	ZYMO Research	D3025
NucleoSpin Gel and PCR Clean-up Mini Kit	Macherey-Nagel	740609.250
NEBNext^®^ Library Quant Kit for Illumina	NEB	E7630
Qubit dsDNA HS assay kit	Invitrogen^™^	Q33231
Chromium Next GEM Single Cell 5’ Reagent Kits v2	10x Genomics	PN-1000263
qScript cDNA SuperMix	VWR	101414–106
Dead cell removal kit	Miltenyi Biotec	130–090-101
Deposited data		
scRNA-seq/Perturb-seq data	This study	GEO: GSE221578
scRNA-seq/scATAC-Seq data	Chen et al.^55^	https://data.humantumoratlas.org/
Healthy donor scRNA-seq	Granja et al.^67^	GEO: GSE139369
ATAC-seq	Corces et al. ^56^	GEO: GSE74912
Pediatric AML H3K27ac ChIP-seq	Perez et al. ^78^	GEO: GSE155558
B-ALL H3K27ac ChIP-seq	Saint Fleur-Lominy et al.^7 9^	GEO: GSE156563
AML H3K27ac CUT&Tag	Xu et al.^80^	GEO: GSE152136
Experimental models: Cell lines		
Human: MOLM-13	DSMZ	ACC-554
Human: HEK293T	ATCC	CRL-3216
Experimental models: Organisms/strains		
NSG	JAX	Stock #: 005557
NSGS	JAX	Strain #:013062
Oligonucleotides		
sgRNA sequences included in the study	This study	Table S4
Recombinant DNA		
LRG2.1	Addgene	108098
SFFV-Cas9–2A-mCherry	Addgene	251397
SFFV-KRAB-dCas9–2A-mCherry	This study	N/A
SFFV-KRAB(W8E)-dCas9–2A-mCherry	This study	N/A
SFFV-ZIM3-dCas9–2A-mCherry	Addgene	251398
dCas9-KRAB-mCherry	Addgene	163956
pLX303-ZIM3-KRAB-dCas9	Addgene	154472
LRG	Addgene	65656
Software and algorithms		
MAGeCK v0.5.0+9	Li et al.^44^	https://sourceforge.net/p/mageck/wiki/Home/
Cell Ranger version 6.1.2	10x Genomics	https://github.com/10XGenomics/cellranger
R version 4.4.0	R Core Team	https://www.r-project.org
FlowJo software, v10.0.7	FlowJo	N/A
Seurat version 4.1.1	Github	https://github.com/satijalab/seurat
msigdb (v 7.1)	GSEA	https://www.gsea-msigdb.org/gsea/msigdb
ggplot2 (v 3.5.1)	R	https://cran.r-project.org/web/packages/ggplot2/index.html
ClusterProfiler (v 4.10.0)	R Bioconductor	https://bioconductor.org/packages/release/bioc/html/clusterProfiler.html
GraphPad Prism 9	GraphPad Software	N/A
Other		
Codes used in this study	This study	Zenodo: 10.5281/zenodo.18176272

## Highlights

- Optimized CRISPR platform for genetic studies in primary AML samples
- *In vitro* and *in vivo* CRISPR screens reveal shared and distinct AML dependencies
- CRISPRi screens identify essential *cis-*regulatory elements in PDXs
- Perturb-seq dissects regulatory networks and heterogeneity in primary AML cells
