# Supplementary material for: CRISPR-based functional genomics for dissecting therapeutic dependency in primary acute myeloid leukemia samples
Source: Mol Cell. Author manuscript; Available in PMC 2026 Mar 31. (PMC13036604; doi:10.1016/j.molcel.2026.02.003)
Supplement: 1 [file NIHMS2146427-supplement-1.pdf]

Supplementary figures

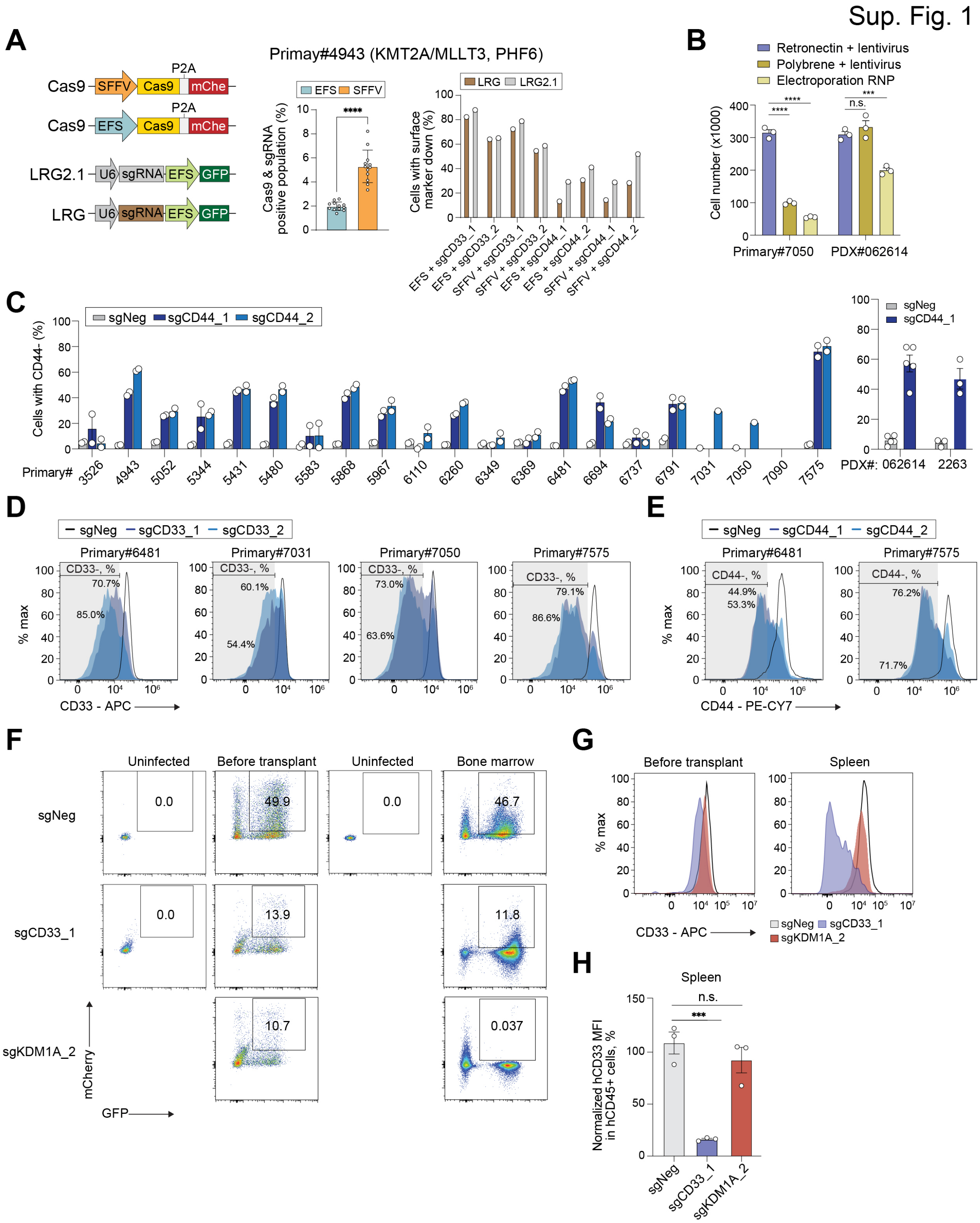

Figure S1 Establishment of a robust CRISPR knockout system in AML PDX and primary patient cells. Related to Figure 1.

(**A**) Configuration of different CRISPR and sgRNA vectors evaluated in primary AML cells (left). Quantification of Cas9 and sgRNA double positive percentage (n = 12) using either EFS- or SFFV-driven Cas9 vector in Primary#4943 cells on day 5 post-transduction (middle). Quantification of the perturbation efficiency of surface markers using either sgRNA-expressing LRG or LRG2.1 vector in Primary#4943 cells on day 7 post-transduction (right). (**B**) Bar graph showing total live cell number on day 3 post-transduction with CRISPR system using the indicated methods in Primary#7050 and PDX#062614 samples (n = 3). *p* values were calculated via unpaired two-tailed t-test. ***, $p < 0.001$, ****, $p < 0.0001$, n.s., not significant. (**C**) Quantification of CRISPR-mediated CD44 perturbation efficiency on day 7 post-transduction with indicated sgRNAs in a panel of AML patient-derived samples, measured by flow cytometry (n = 3–5 for PDX samples, n = 2 for primary AML cells). (**D**) Representative flow cytometry plots of CD33 expression in Cas9+/sgRNA+ (mChe+/GFP+) Primary#6481, Primary#7031, Primary#7050 and Primary#7575 (from left to right) cells with indicated sgRNA on day 7 post-transduction. (**E**) Representative flow cytometry plots of CD44 expression in Cas9+/sgRNA+ (mChe+/GFP+) Primary#6481 (left) and Primary#7575 (right) cells with indicated sgRNA on day 7 post-transduction. (**F**) Flow cytometry analysis of GFP and mCherry expression in PDX-2263 cells transduced with Cas9 and indicated sgRNA. Left panel shows samples before transplant for *in vivo* experiments, 3 days post-transduction of sgRNA. Right panel shows bone marrow from leukemic mice. (**G**) Representative flow cytometry of CD33 expression in PDX#2263 cells before transplant for *in vivo* experiments, 3 days post-transduction of sgRNA (left) and in Cas9+/sgRNA+/hCD45+ PDX#2263 cells with indicated sgRNA in spleen (right). (**H**) Quantification of CD33 expression in Cas9+/sgRNA+/hCD45+ PDX#2263 cells with indicated sgRNA in SP (n=2-4). *p* values were calculated via unpaired two-tailed t-test. ***, $p < 0.001$. n.s., not significant. All error bars represent the mean ± SEM.

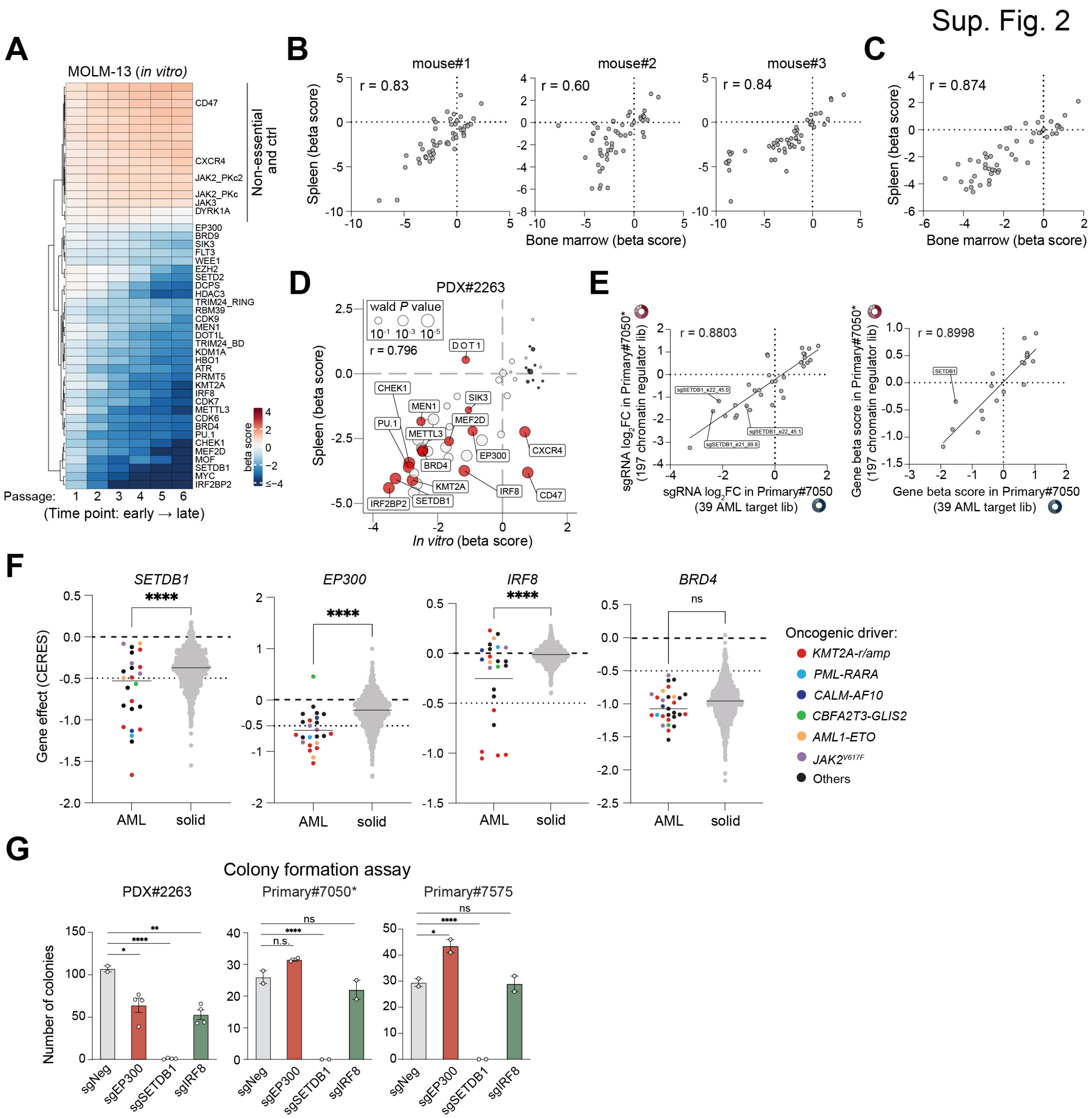
Sup. Fig. 2
A
MOLM-13 (in vitro)
Non-essential and ctrl
beta score
Passage: 1 2 3 4 5 6
(Time point: early → late)
B
mouse#1
mouse#2
mouse#3
r = 0.83
r = 0.60
r = 0.84
Spleen (beta score)
Bone marrow (beta score)
C
r = 0.874
D
PDX#2263
wald P value
r = 0.796
Spleen (beta score)
In vitro (beta score)
E
r = 0.8803
r = 0.8998
sgRNA log2FC in Primary#7050* (197 chromatin regulator lib)
sgRNA log2FC in Primary#7050 (39 AML target lib)
Gene beta score in Primary#7050* (197 chromatin regulator lib)
Gene beta score in Primary#7050 (39 AML target lib)
F
SETDB1
EP300
IRF8
BRD4
Gene effect (CERES)
AML
solid
Oncogenic driver:
KMT2A-r/amp
PML-RARA
CALM-AF10
CBFA2T3-GLIS2
AML1-ETO
JAK2V617F
Others
G
Colony formation assay
PDX#2263
Primary#7050*
Primary#7575
Number of colonies
sgNeg
sgEP300
sgSETDB1
sgIRF8

Figure S2. CRISPR knockout dropout screens in AML PDX and primary patient samples revealed shared and distinct dependencies. Related to Figure 2.

(**A**) An *in vitro* pilot screen using the “39 AML target” library in MOLM-13 cells. Transduced cells were collected at 6 different time points, and the heatmap depicts the hierarchical clustering of CRISPR beta scores for each target. (**B**) Scatterplots showing correlations of target gene beta scores between bone marrow and spleen from the 3 individual mice. (**C**) Scatterplots showing correlations of the median target gene beta scores derived from **B**). (**D**) Scatterplots showing correlations of target gene beta score from CRISPR screens using 39 “AML target” library between *in vitro* and spleen in PDX#2263 cells. Black circles represent negative control sgRNAs spiked into the libraries, while red circles denote shared and distinct gene dependencies as described in the text. Circle size corresponds to –log10(Wald p-value) from the MAGeCK-MLE module, with larger circles indicating greater statistical significance. (**E**) Scatterplots showing correlations of shared sgRNAs between the 39 AML target library and the 197 chromatin regulator library screens in Primary#7050 and Primary#7050* cells. Correlations are shown for individual sgRNA $\log_2$(fold change) values (left) and target gene beta scores (right). (**F**) Comparison of *SETDB1*, *EP300*, *IRF8* and *BRD4* dependencies between AML and solid tumor cell lines from Depmap 22Q2 public+score dataset. Plotted is the relative sgRNA+/Cas9+ population normalized to the one on day 3 or 4. ****, $p<0.0001$. *p* values were calculated via unpaired two-tailed t-test. (**G**) Quantification of colony formation assay with indicated sgRNAs in Cas9-expressing PDX#2263 (left), primary#7050*(middle) and primary#7575 (right) cells. *p* values were calculated via unpaired two-tailed t-test. *, $p <0.1$, **, $p <0.01$, ****, $p <0.0001$. n.s., not significant. All error bars represent the mean ± SEM.

Sup. Fig. 3

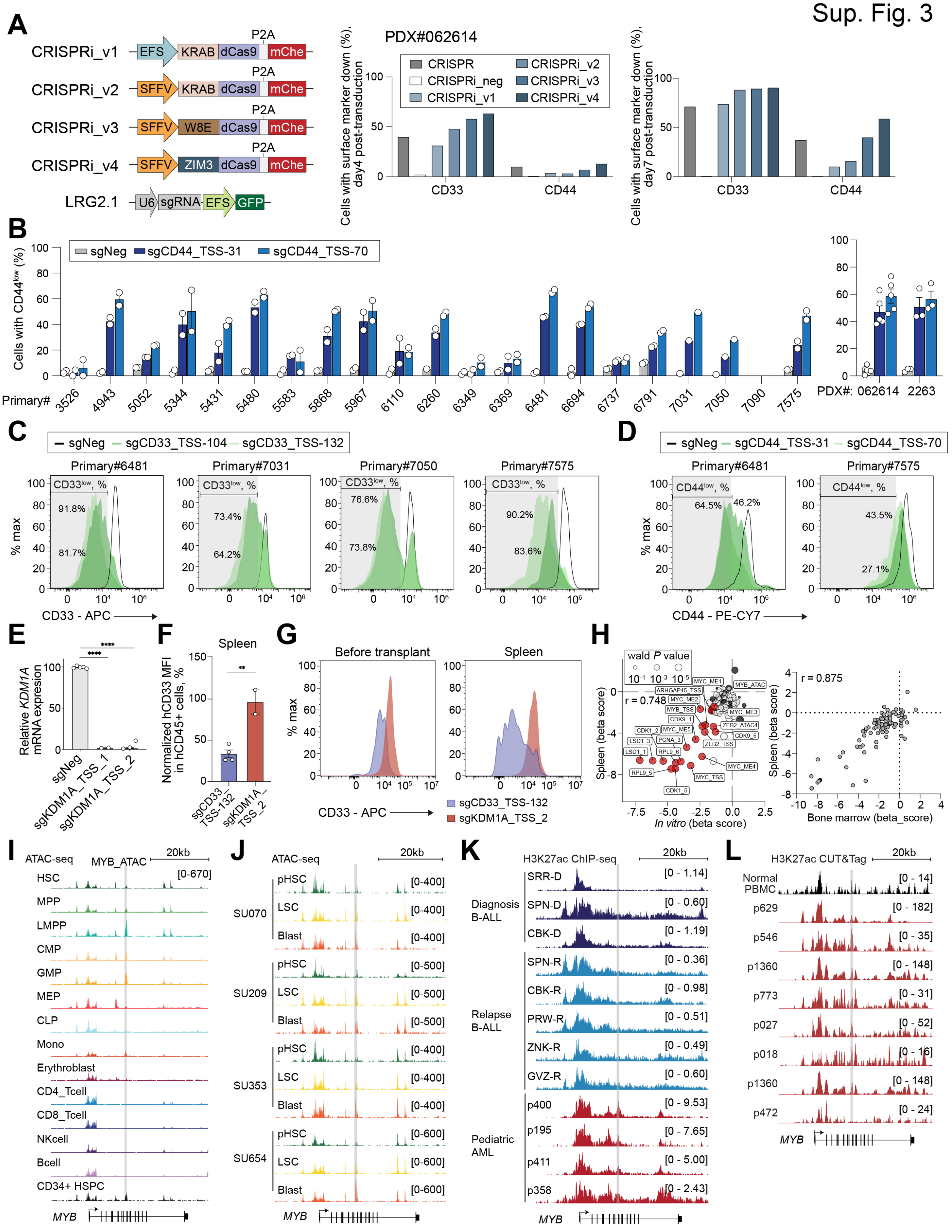
A
CRISPRi_v1
CRISPRi_v2
CRISPRi_v3
CRISPRi_v4
LRG2.1
EFS KRAB dCas9 P2A mChe
SFFV KRAB dCas9 P2A mChe
SFFV W8E dCas9 P2A mChe
SFFV ZIM3 dCas9 P2A mChe
U6 sgRNA EFS GFP
PDX#062614
CRISPR
CRISPRi_neg
CRISPRi_v1
CRISPRi_v2
CRISPRi_v3
CRISPRi_v4
Cells with surface marker down (%), day4 post-transduction
Cells with surface marker down (%), day7 post-transduction
CD33
CD44
B
sgNeg
sgCD44_TSS-31
sgCD44_TSS-70
Cells with CD44low (%)
Primary#
PDX#: 062614 2263
C
sgNeg
sgCD33_TSS-104
sgCD33_TSS-132
Primary#6481
Primary#7031
Primary#7050
Primary#7575
% max
CD33 - APC
D
sgNeg
sgCD44_TSS-31
sgCD44_TSS-70
CD44 - PE-CY7
E
Relative KDM1A mRNA expression
sgNeg
sgKDM1A_TSS_1
sgKDM1A_TSS_2
F
Spleen
Normalized hCD33 MFI in hCD45+ cells, %
sgCD33_TSS-132
sgKDM1A_TSS_2
G
Before transplant
Spleen
CD33 - APC
sgCD33_TSS-132
sgKDM1A_TSS_2
H
wald P value
r = 0.748
r = 0.875
Spleen (beta score)
In vitro (beta score)
Bone marrow (beta_score)
I
ATAC-seq
MYB_ATAC
20kb
HSC
MPP
LMPP
CMP
GMP
MEP
CLP
Mono
Erythroblast
CD4_Tcell
CD8_Tcell
NKcell
Bcell
CD34+ HSPC
MYB
J
ATAC-seq
SU070
SU209
SU353
SU654
pHSC
LSC
Blast
K
H3K27ac ChIP-seq
Diagnosis B-ALL
Relapse B-ALL
Pediatric AML
SRR-D
SPN-D
CBK-D
SPN-R
CBK-R
PRW-R
ZNK-R
GVZ-R
p400
p195
p411
p358
L
H3K27ac CUT&Tag
Normal PBMC
p629
p546
p1360
p773
p027
p018
p1360
p472

Figure S3. CRISPRi knockdown genetic screens identified functional cis-regulatory elements in AML PDXs. Related to Figure 3.

(**A**) Configuration of different CRISPRi vectors evaluated in AML PDX cells (left). Quantification of the perturbation efficiency of either CD33 or CD44 expression using indicated CRISPRi vectors in PDX#062614 cells on day 7 post-transduction (right). (**B**) Quantification of the perturbation efficiency of CD44 by CRISPRi in primary AML samples (n = 2) (left) and PDX cells (n = 3-6) (right) on day 7 post-transduction. (**C**) Representative flow cytometry plots of CD33 expression in dCas9-ZIM3+/sgRNA+ Primary#6481, Primary#7031, Primary#7050, and Primary#7575 (from left to right) cells with indicated sgRNA on day 7 post-transduction. (**D**) Representative flow cytometry plots of CD44 expression in dCas9-ZIM3+/sgRNA+ Primary#6481 (left) and primary#7575 (right) cells with indicated sgRNA on day 7 post-transduction. (**E**) qPCR quantification of relative *KDM1A* mRNA level in MOLM-13 cells transduced with ZIM3-dCas9 and indicated sgRNAs (n = 2-3). *p* values were calculated via unpaired two-tailed t-test. ****, p <0.0001. (**F**) Quantification of CD33 expression in dCas9-ZIM3+/sgRNA+ hCD45+ PDX#2263 cells with indicated sgRNA in spleen (n = 2-4). *p* values were calculated via unpaired two-tailed t-test. **, p <0.01.(**G**) Representative flow cytometry of CD33 expression in dCas9-ZIM3+/sgRNA+ hCD45+ PDX#2263 cells with indicated sgRNA in spleen (left) and in PDX-2263 cells before transplant for *in vivo* experiments, 3 days post-transduction of sgRNA (right). (**H**) Scatterplots showing correlations of target beta scores between *in vitro* and spleen (left), as well as between bone marrow and spleen (right) in PDX#2263 cells. Black circles represent negative control sgRNAs spiked into the libraries, while red circles denote known and dependencies as described in the text. Circle size corresponds to –log10(Wald p-value) from the MAGeCK-MLE module, with larger circles indicating greater statistical significance. (**I**) Genome browser tracks of ATAC-seq signals at *MYB* locus in normal human hematopoiesis[66]. (**J**) Genome browser tracks of ATAC-seq signals at *MYB* locus during AML evolution. pHSC, preleukemic hematopoietic stem cell; LSC, a leukemic stem cell. Each set of pHSC, LSC and blasts was sorted from the same patient[66]. (**K**) Genome browser tracks of H3K27ac ChIP-seq signals at *MYB* locus in childhood diagnosis (dark blue) and the relapsed (light blue) childhood B-ALL pairs and pediatric AML (red)[78, 79]. (**L**) Genome browser tracks of H3K27ac CUT&Tag (right) signals at the MYB locus in adult AML patients[80]. All error bars indicate mean ± SEM.

Sup. Fig. 4

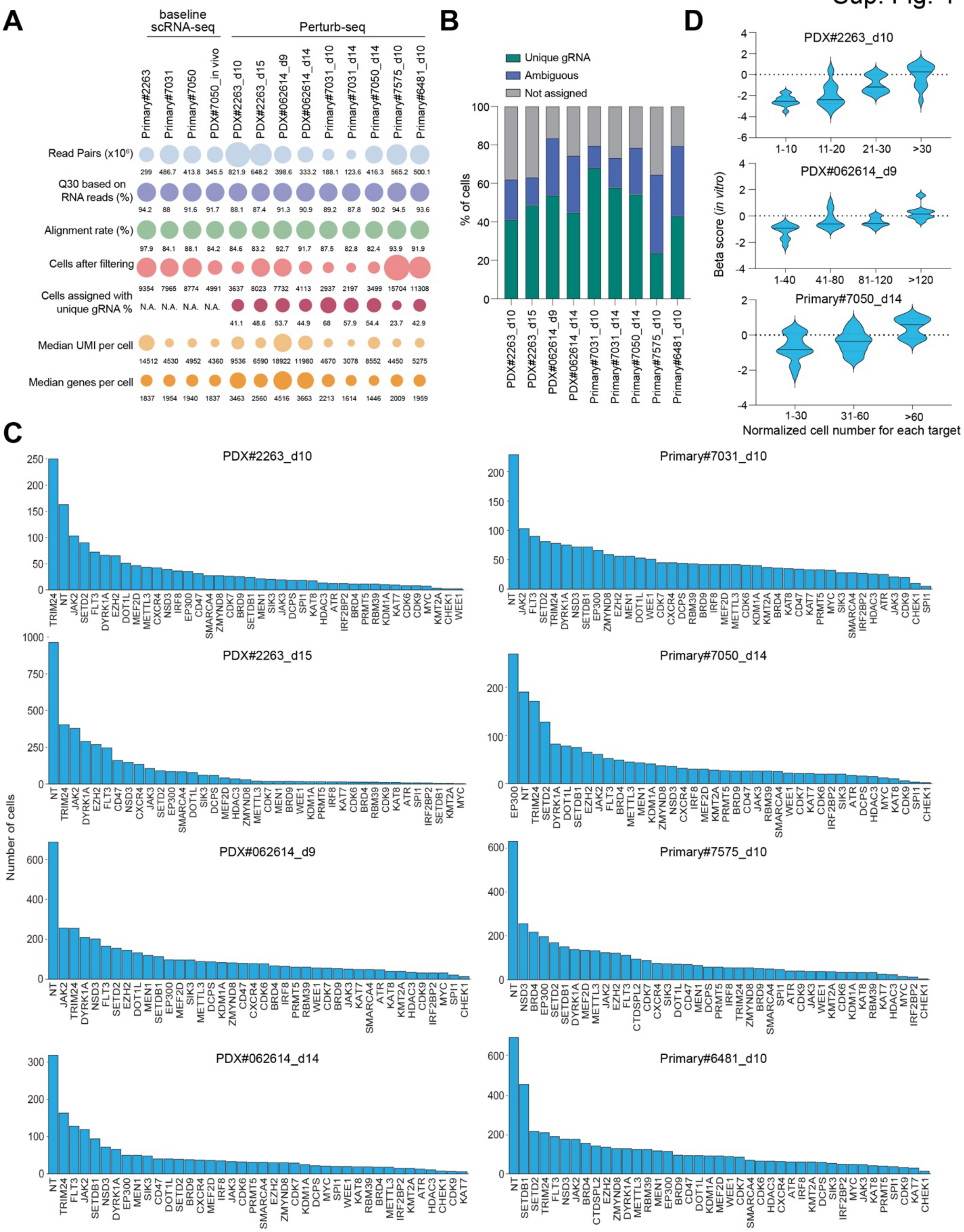
A
baseline scRNA-seq
Perturb-seq
Primary#2263
Primary#7031
Primary#7050
PDX#7050_in vivo
PDX#2263_d10
PDX#2263_d15
PDX#062614_d9
PDX#062614_d14
Primary#7031_d10
Primary#7031_d14
Primary#7050_d14
Primary#7575_d10
Primary#6481_d10
Read Pairs (x10⁶)
299 486.7 413.8 345.5 821.9 648.2 398.6 333.2 188.1 123.6 416.3 565.2 500.1
Q30 based on RNA reads (%)
94.2 88 91.6 91.7 88.1 87.4 91.3 90.9 89.2 87.8 90.2 94.5 93.6
Alignment rate (%)
97.9 84.1 88.1 84.2 84.6 83.2 92.7 91.7 87.5 82.8 82.4 93.9 91.9
Cells after filtering
9354 7965 8774 4991 3637 8023 7732 4113 2937 2197 3499 15704 11308
Cells assigned with unique gRNA %
N.A. N.A. N.A. N.A. 41.1 48.6 53.7 44.9 68 57.9 54.4 23.7 42.9
Median UMI per cell
14512 4530 4952 4360 9536 6590 18922 11980 4670 3078 8552 4450 5275
Median genes per cell
1837 1954 1940 1837 3463 2560 4516 3663 2213 1614 1446 2009 1959
B
Unique gRNA
Ambiguous
Not assigned
% of cells
D
PDX#2263_d10
1-10 11-20 21-30 >30
PDX#062614_d9
1-40 41-80 81-120 >120
Primary#7050_d14
1-30 31-60 >60
Beta score (in vitro)
Normalized cell number for each target
C
Number of cells
PDX#2263_d10
PDX#2263_d15
PDX#062614_d9
PDX#062614_d14
Primary#7031_d10
Primary#7050_d14
Primary#7575_d10
Primary#6481_d10

Figure S4. Perturb-seq of AML patient cells revealed regulators of genes related to leukemia maintenance. Related to Figure 4.

(**A**) Summary statistics of all scRNA-seq and Perturb-seq data. (**B**) Detection of sgRNA from the single-cell transcriptome data. (**C**) The number of cells assigned to a unique target in the Perturb-seq data of indicated PDX and primary AML samples. (**D**) Violin plots showing correlation between beta scores and cell numbers of target gene.

Sup. Fig. 5

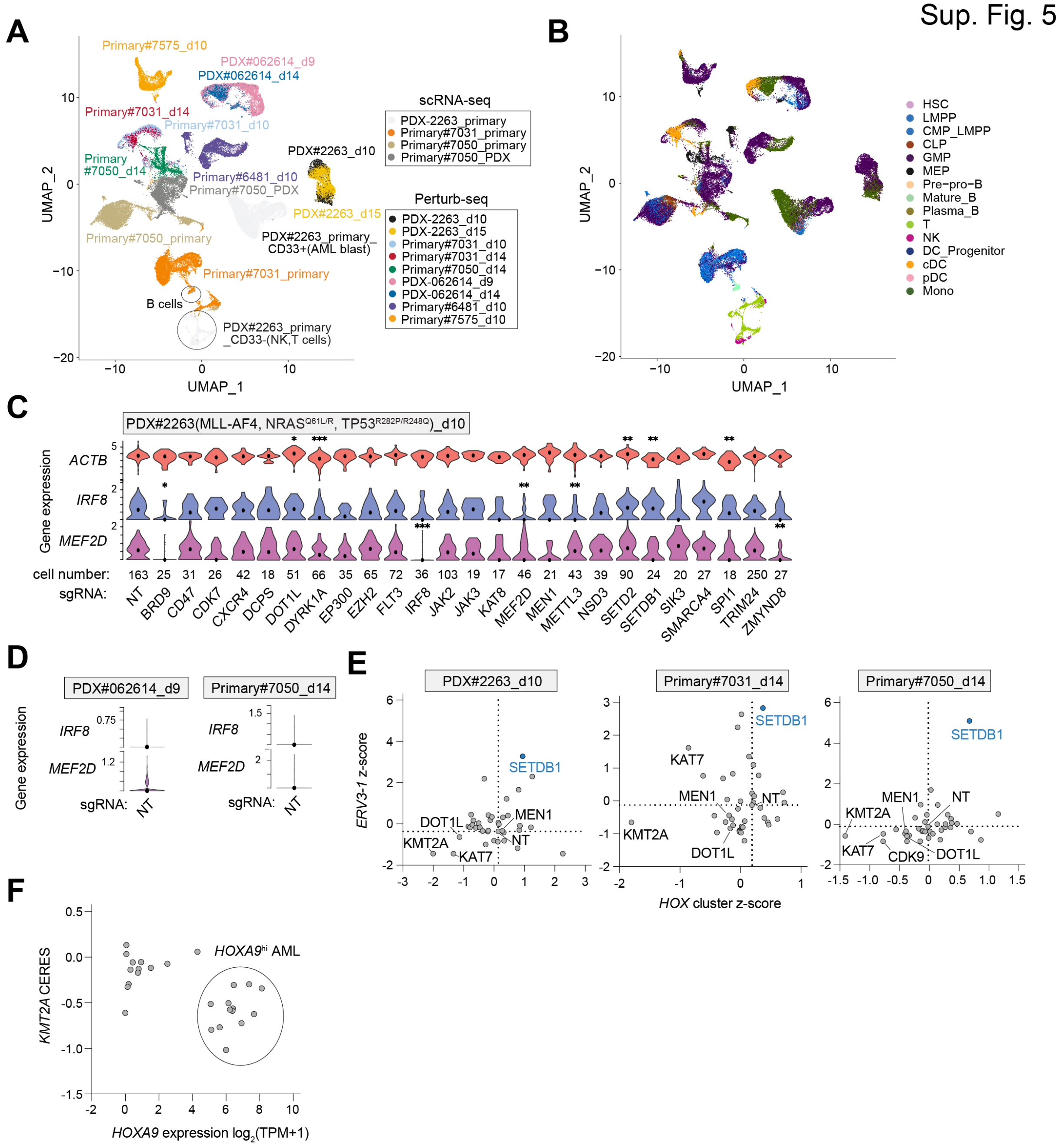

A
Primary#7575_d10
PDX#062614_d9
PDX#062614_d14
Primary#7031_d14
Primary#7031_d10
Primary #7050_d14
Primary#6481_d10
Primary#7050_PDX
PDX#2263_d10
PDX#2263_d15
Primary#7050_primary
PDX#2263_primary_ CD33+(AML blast)
Primary#7031_primary
B cells
PDX#2263_primary _CD33-(NK,T cells)
UMAP_1
UMAP_2
scRNA-seq
PDX-2263_primary
Primary#7031_primary
Primary#7050_primary
Primary#7050_PDX
Perturb-seq
PDX-2263_d10
PDX-2263_d15
Primary#7031_d10
Primary#7031_d14
Primary#7050_d14
PDX-062614_d9
PDX-062614_d14
Primary#6481_d10
Primary#7575_d10
B
HSC
LMPP
CMP_LMPP
CLP
GMP
MEP
Pre−pro−B
Mature_B
Plasma_B
T
NK
DC_Progenitor
cDC
pDC
Mono
C
PDX#2263(MLL-AF4, NRAS Q61L/R, TP53 R282P/R248Q)_d10
Gene expression
ACTB
IRF8
MEF2D
cell number: 163 25 31 26 42 18 51 66 35 65 72 36 103 19 17 46 21 43 39 90 24 20 27 18 250 27
sgRNA: NT BRD9 CD47 CDK7 CXCR4 DCPS DOT1L DYRK1A EP300 EZH2 FLT3 IRF8 JAK2 JAK3 KAT8 MEF2D MEN1 METTL3 NSD3 SETD2 SETDB1 SIK3 SMARCA4 SPI1 TRIM24 ZMYND8
D
PDX#062614_d9
Primary#7050_d14
IRF8
MEF2D
sgRNA: NT
E
PDX#2263_d10
Primary#7031_d14
Primary#7050_d14
ERV3-1 z-score
HOX cluster z-score
SETDB1
DOT1L
KMT2A
KAT7
MEN1
NT
CDK9
F
KMT2A CERES
HOXA9 expression log2(TPM+1)
HOXA9hi AML

Figure S5. Perturb-seq of AML patient cells revealed regulators of genes related to leukemia maintenance. Related to Figure 4.

(**A**) Uniform manifold approximation and projection **(**UMAP) visualization of all patients' scRNA-seq and Perturb-seq data. (**B**) UMAP visualization of all patients' scRNA-seq and Perturb-seq data, projected to healthy BM reference to implicate the cell types. (**C**) Violin plots showing *ACTB* (housekeeping), *IRF8,* and *MEF2D* expression upon perturbations (column) in PDX#2263 cells at day 10 post-transduction of indicated sgRNAs. Cell numbers for each target gene are shown as indicated. *p* values were calculated via Wilcoxon rank-sum test followed by multiple hypothesis test correction. *, p<0.05, **, p <0.01, ***, p<0.001. (**D**) Violin plots showing *IRF8* and *MEF2D* expression of the NT negative control in PDX-062614 at day 9 post-transduction of sgRNA (left) and primary#7050 at day 14 post-transduction of sgRNA (right). (**E**) The perturbation effect of the indicated sgRNAs on the *HOX* cluster (x-axis) and *ERV3-1* expression (y-axis) was analyzed in PDX#2263 at day 10 post-transduction, Primary#7031 at day 14 post-transduction and Primary#7050 at day 14 post-transduction (from left to right), with the perturbation effects being indicated by z-statistic scores. (**F**) *KMT2A* dependencies in AML cells with low and high (circle) *HOXA9* expression. Data retrieved from Depmap 22Q2 public + score dataset.

Sup. Fig. 6

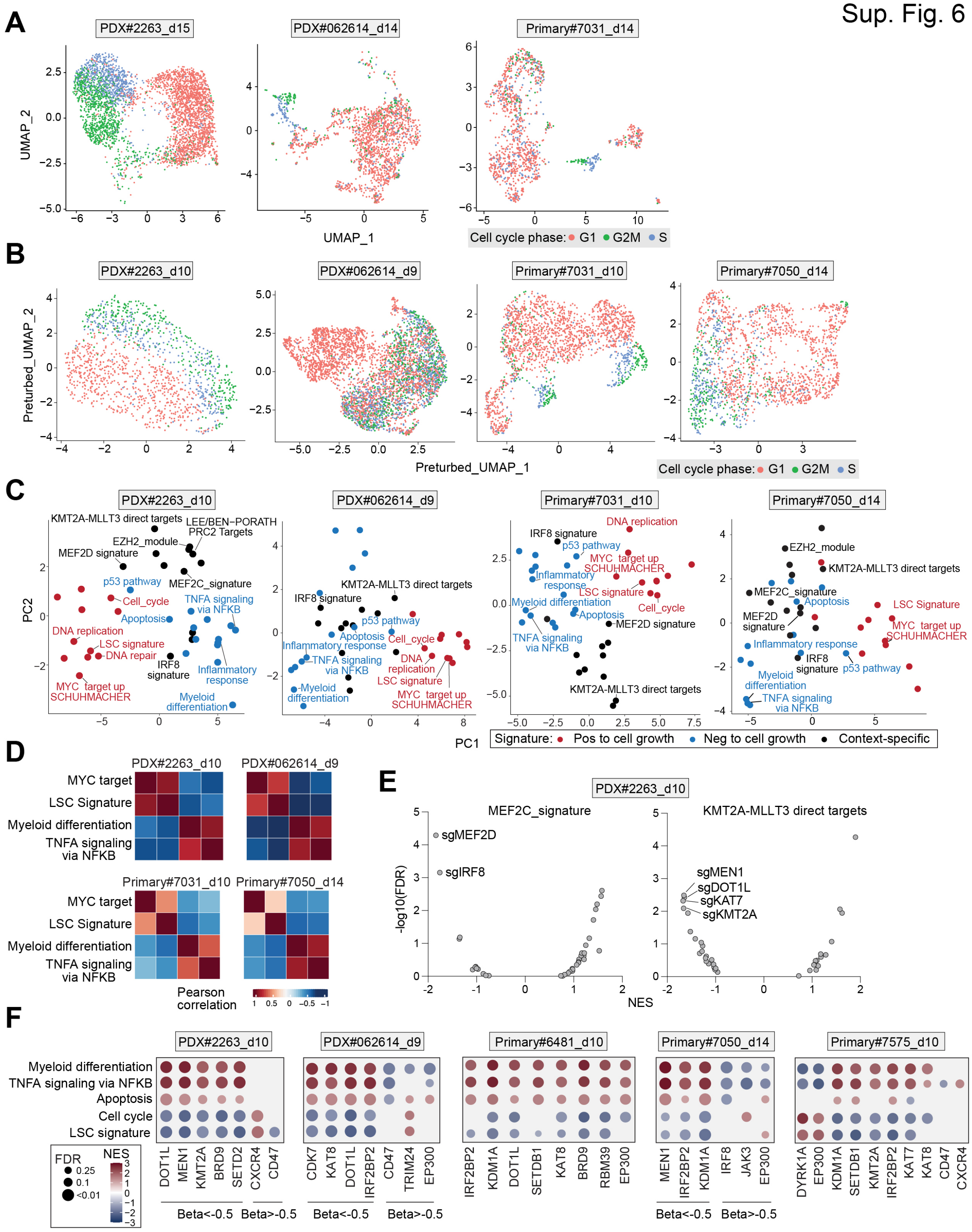
A
PDX#2263_d15
PDX#062614_d14
Primary#7031_d14
UMAP_2
UMAP_1
Cell cycle phase: G1 G2M S
B
PDX#2263_d10
PDX#062614_d9
Primary#7031_d10
Primary#7050_d14
Preturbed_UMAP_2
Preturbed_UMAP_1
Cell cycle phase: G1 G2M S
C
PDX#2263_d10
PDX#062614_d9
Primary#7031_d10
Primary#7050_d14
PC2
PC1
KMT2A-MLLT3 direct targets
LEE/BEN−PORATH PRC2 Targets
EZH2_module
MEF2D signature
MEF2C_signature
p53 pathway
Cell_cycle
TNFA signaling via NFKB
Apoptosis
DNA replication
LSC signature
DNA repair
IRF8 signature
Inflammatory response
MYC target up SCHUHMACHER
Myeloid differentiation
Signature: Pos to cell growth Neg to cell growth Context-specific
D
MYC target
LSC Signature
Myeloid differentiation
TNFA signaling via NFKB
Pearson correlation
E
PDX#2263_d10
MEF2C_signature
KMT2A-MLLT3 direct targets
-log10(FDR)
NES
sgMEF2D
sgIRF8
sgMEN1
sgDOT1L
sgKAT7
sgKMT2A
F
PDX#2263_d10
PDX#062614_d9
Primary#6481_d10
Primary#7050_d14
Primary#7575_d10
Myeloid differentiation
TNFA signaling via NFKB
Apoptosis
Cell cycle
LSC signature
FDR
0.25
0.1
<0.01
NES
DOT1L MEN1 KMT2A BRD9 SETD2 CXCR4 CD47
CDK7 KAT8 DOT1L IRF2BP2 CD47 TRIM24 EP300
IRF2BP2 KDM1A DOT1L SETDB1 KAT8 BRD9 RBM39 EP300
MEN1 IRF2BP2 KDM1A IRF8 JAK3 EP300
DYRK1A EP300 KDM1A SETDB1 KMT2A IRF2BP2 KAT7 KAT8 CD47 CXCR4
Beta<-0.5 Beta>-0.5

Figure S6. Perturb-seq analysis of proliferation-associated gene signatures predicted target essentiality in AML. Related to Figure 5.

(**A**) UMAP plot of individual cell cycle phases analyzed from the indicated Perturb-seq samples. (**B**) UMAP plot of individual cell cycle phases analyzed from the indicated Perturb-seq samples after applying the Mixscape package[7] to mitigate the cell cycle effect. (**C**) Principal component analysis (PCA) analysis of normalized enrichment score (NES) of Gene Set Enrichment Analysis (GSEA) of indicated samples. Leukemia-essential gene sets were analyzed using a refined gene set list, which includes 11 gene sets generally associated with positive regulators of cell growth (e.g., cell cycle, LSC, MYC-target gene sets, labeled in red), 14 gene sets associated with negative regulators of cell growth (e.g., myeloid differentiation, apoptosis, inflammatory pathways, marked in blue), and 13 gene sets that are either neutral or context-specific (labeled in dark gray). NES was calculated by comparing the scRNA-seq data of each target gene to the NT control group. (**D**) Heatmaps depicting Pearson correlation of NESs of indicated gene sets in Perturb-seq data of indicated PDX and primary samples. (**E**) Dot plots showing GSEA analysis of the MEF2C_signature (left) and the KMT2A-MLLT3 direct targets (right) in Perturb-seq data of PDX#2263 at day 10 post-transduction. (**F**) Bubble plots showing examples of GSEA results upon indicated perturbations in indicated PDX and primary samples. *p* values were calculated via unpaired two-tailed t-test.

Sup. Fig. 7

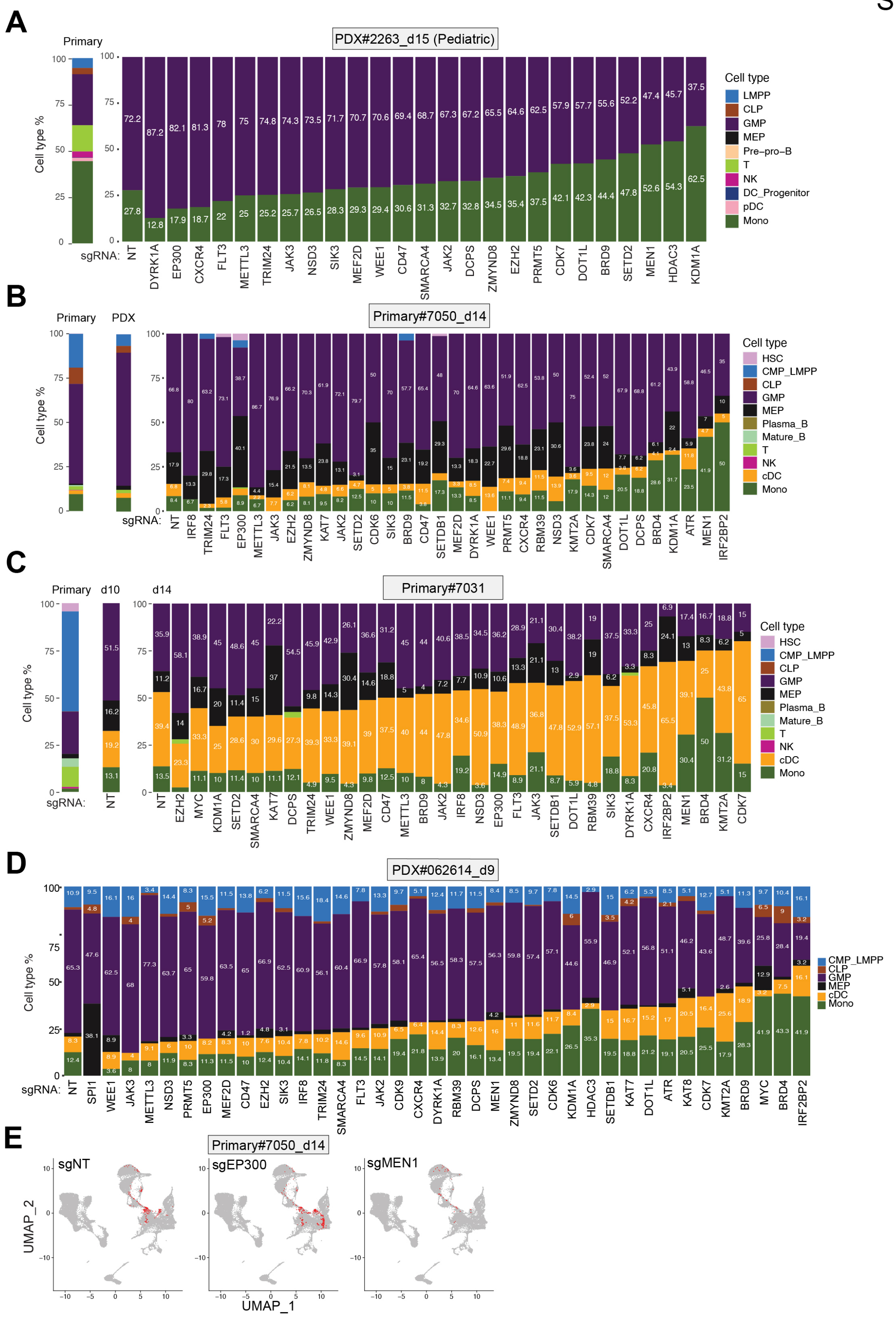
A
Primary
PDX#2263_d15 (Pediatric)
Cell type %
Cell type
LMPP
CLP
GMP
MEP
Pre–pro–B
T
NK
DC_Progenitor
pDC
Mono
sgRNA:
NT DYRK1A EP300 CXCR4 FLT3 METTL3 TRIM24 JAK3 NSD3 SIK3 MEF2D WEE1 CD47 SMARCA4 JAK2 DCPS ZMYND8 EZH2 PRMT5 CDK7 DOT1L BRD9 SETD2 MEN1 HDAC3 KDM1A
B
Primary
PDX
Primary#7050_d14
Cell type %
Cell type
HSC
CMP_LMPP
CLP
GMP
MEP
Plasma_B
Mature_B
T
NK
cDC
Mono
sgRNA:
NT IRF8 TRIM24 FLT3 EP300 METTL3 JAK3 EZH2 ZMYND8 KAT7 JAK2 SETD2 CDK6 SIK3 BRD9 CD47 SETDB1 MEF2D DYRK1A WEE1 PRMT5 CXCR4 RBM39 NSD3 KMT2A CDK7 SMARCA4 DOT1L DCPS BRD4 KDM1A ATR MEN1 IRF2BP2
C
Primary
d10
d14
Primary#7031
Cell type %
Cell type
HSC
CMP_LMPP
CLP
GMP
MEP
Plasma_B
Mature_B
T
NK
cDC
Mono
sgRNA:
NT
NT EZH2 MYC KDM1A SETD2 SMARCA4 KAT7 DCPS TRIM24 WEE1 ZMYND8 MEF2D CD47 METTL3 BRD9 JAK2 IRF8 NSD3 EP300 FLT3 JAK3 SETDB1 DOT1L RBM39 SIK3 DYRK1A CXCR4 IRF2BP2 MEN1 BRD4 KMT2A CDK7
D
PDX#062614_d9
Cell type %
CMP_LMPP
CLP
GMP
MEP
cDC
Mono
sgRNA:
NT SPI1 WEE1 JAK3 METTL3 NSD3 PRMT5 EP300 MEF2D CD47 EZH2 SIK3 IRF8 TRIM24 SMARCA4 FLT3 JAK2 CDK9 CXCR4 DYRK1A RBM39 DCPS MEN1 ZMYND8 SETD2 CDK6 KDM1A HDAC3 SETDB1 KAT7 DOT1L ATR KAT8 CDK7 KMT2A BRD9 MYC BRD4 IRF2BP2
E
Primary#7050_d14
sgNT
sgEP300
sgMEN1
UMAP_2
UMAP_1

Figure S7. Perturb-seq projection onto normal hematopoietic trajectory revealed target gene impact on cellular composition. Related to Figure 6.

(**A**) Projection of scRNA-seq data from the original stock of primary counterpart of PDX#2263 (left) and Perturb-seq data from PDX-2263 at day 15 post-transduction of the indicated sgRNA (right) onto the healthy pediatric reference UMAP reconstructed in **Figure 6A**. The sgRNAs were ranked in descending order by the percentage of the progenitor and stem cell population, with sgNT on the left side. sgRNAs with representation from at least 15 cells in the Perturb-seq data were included in the analysis. The percentage of each cell population is labeled in the bar. (**B**) Projection of scRNA-seq data from the original stock of Primary#7050 sample (left) and PDX counterpart of Primary#7050 (middle) to healthy adult reference UMAP reconstructed in **Figure 6B**. Full data for **Figure 6G** (right). (**C**) Projection of scRNA-seq data from the original stock of Primary#7031 sample (left), Perturb-seq data from Primary#7031 at day 10 post-transduction of the sgNT (middle) to healthy adult reference UMAP reconstructed in **Figure 6B**. Full data for **Figure 6F** (right). (**D**) Full data for **Figure 6D** (left). (**F**) Projection of Perturb-seq data from Primary#7050 at day 14 post-transduction of the indicated sgRNA, sgNT (left), sgEP300 (middle), and sgMEN1 (right), onto the healthy adult reference UMAP reconstructed in **Figure 6B**.

Methods S1. Detailed primary AML virus transduction protocol, related to STAR Methods.

Making lentivirus

1. **Day before transfection:** Plate HEK293T cells in DMEM (10% FBS, 1% P/S) in a 10-cm plate in the afternoon.
    a. Vacuum media off of a >95% confluent plate of 293T cells
    b. Gently add 1 mL of 1X PBS to wash the cells
    c. Vacuum off 1X PBS
    d. Add 1 mL of 1X Trypsin
    e. Incubate at 37°C for ~5 min
    f. Add 9 mL of DMEM to resuspend the cells
    g. For each virus plate, add 5mL of 293T cells + 5mL DMEM
    h. Incubate O/N at 37°C
2. **Morning of Day 0:** Check cells to make sure that they are ~90-100% confluent. If so, follow steps a-g. If not, wait another day to start.
    a. Thaw DNA plasmids, VSVG, and psPAX2
    b. Get OPTI-MEM and PEI from cold room. Vortex and spin down the PEI
    c. For Each virus you will have a ‘Tube A’ and a ‘Tube B’
        i. Tube A:
            1. Combine 500 µl of OPTI-MEM and 80 µl of PEI.
            2. Vortex and incubate in the TC hood at RT for 5 min.
        ii. Tube B:
            1. While Tube A is incubating, combine 500 µl of OPTI-MEM, 10 µg of Lenti plasmid, 5 µg of VSVG, and 7.5 µg of psPAX2 in Tube B.
    d. Add all of Tube A into Tube B
    e. Vortex Tube B for ~15 seconds and briefly spin down
    f. Incubate Tube B at RT in the TC hood for ~18 minutes.
    g. After ~18 mins, gently drop the solution onto your cells. Swirl the plate and place in 37°C incubator.
3. **Afternoon of Day 0:** Change the media on the cells after 6-8 hours.
    a. Gently vacuum off media. (It is good to leave a bit on the cells so that they do not peel off when you replace the media).
    b. Tilt the front of the plate towards you and gently add ~6mL of fresh DMEM to the edge of the plate. Be very gentle, the cells are sick and extremely confluent so it is easy to disturb them.
    c. Place back in 37°C incubator O/N
4. **Day 1:** Collecting virus.
    a. To collect the virus-containing media, gently tilt the front of the plate towards you and pipet off ~5mL.
    b. Collect in a 50 mL conical tube.
    c. Gently replace media with 5mL of fresh DMEM.
    d. Place cells back in incubator.
    e. Store 50 mL conical in cold room.
    f. 6-7 hours after the first collection, you can collect again.

   g. Repeat steps 4a-4f for 3 days with 2 collections per day.

5. **Day 4:**
   a. Spin down the collected virus at 3000 rpm for 5 min at RT.
   b. Transfer supernatant to a new conical tube.
   c. Filter the supernatant using a 0.45 μm PVDF filter. The supernatant is ready for ultracentrifuge concentration.

Concentrating lentivirus by ultracentrifugation

1. Adding 4ml of 20% sucrose solution to the bottom of each ultracentrifuge tube.
2. Slowly load the filtered lentivirus supernatant (~32ml) on top of the sucrose cushion, without disturbing the sucrose layer.
3. Balance the ultracentrifuge tubes precisely (<0.01g).
4. Spin down the lentivirus in ultracentrifuge at 25000rpm at 4°C for 2.5h.
5. After the spin is done, carefully aspirate supernatant and sucrose without disturbing the pellet.
6. Invert the tubes on sterile paper towel for 5 mins and carefully wipe out the remaining media on the side of the tubes.
7. Resuspend the pellet with one tenth of the supernatant volume 1X PBS (3.2ml PBS for 32ml lentivirus supernatant). Aliquot the concentrated lentivirus and store at -80°C.

Concentrating lentivirus by PEG or Lenti-X Concentrator

1. Protocol adapted from (https://www.mdanderson.org/documents/core-facilities/Functional%20Genomics%20Core/Homemade%204fold%20lentivirus%20concentrator.pdf). Dissolve 80g PEG-8000 (Sigma P5413), 14.0g NaCl in 80ml MillQ water and 20ml of 10×PBS (pH7.4), mix with gentle stirring, and autoclave. The stock solution is 40% (w/v).

2. Add 1 volume of the PEG to 3 volumes of virus supernatant or Lenti-X concentrator, mix well by inverting the tubes several times, and incubate overnight at 4°C.

3. Spin down at 1600xg for 10min at 4°C.

4. Carefully remove the supernatant and resuspend the viral pellet with 1/10 to 1/50 of the original volume of PBS by gently pipetting up and down.

5. Aliquot the concentrated lentivirus and store at -80°C.

Transducing primary AML cells

1. Make Retronectin solution by resuspending 240ul 1ug/ul Retronectin stock in 12ml PBS.
2. Add 1ml Retronectin solution per well in a non-treated 12-well plate. Incubate for 2h at RT or 4°C overnight.
3. After incubation, collect Retronectin solution (can be reused up to 5 times) and block the plate with 1ml of 2%BSA per well in a 12-well plate for 30min at RT.
4. Aspirate BSA, wash with 1ml PBS.
5. To enrich the virus on the plate, pre-spin concentrated virus supernatant (1ml for a 12-well plate) for 1h at 37°C.
6. Count cells for transduction (approximately 500,000 cells/well of a 12-well plate).
7. Aspirate the virus supernatant for enrichment and mix 1ml concentrated virus with 1ml

fresh media with 500,000 cells. Add LentiBlast Premium (2ul per ml).

8. Spin at 2000rpm for 1h at 37°C.
9. Incubate at 37°C for 48h.
10. After incubation, resuspend and spin down the cells at 1500rpm for 5min. Aspirate the supernatant and resuspend the cells in fresh media.
11. Check GFP and/or mCherry percentage 72h post-transduction.
